# Metabolic capabilities are highly conserved among human nasal-associated *Corynebacterium* species in pangenomic analyses

**DOI:** 10.1128/msystems.01132-24

**Published:** 2024-11-07

**Authors:** Tommy H. Tran, Isabel F. Escapa, Ari Q. Roberts, Wei Gao, Abiola C. Obawemimo, Julia A. Segre, Heidi H. Kong, Sean Conlan, Matthew S. Kelly, Katherine P. Lemon

**Affiliations:** 1 Alkek Center for Metagenomics & Microbiome Research, Department of Molecular Virology & Microbiology, Baylor College of Medicine, Houston, Texas, USA; 2 The Forsyth Institute (Microbiology), Cambridge, Massachusetts, USA; 3 Department of Oral Medicine, Infection and Immunity, Harvard School of Dental Medicine, Boston, Massachusetts, USA; 4 Microbial Genomics Section, Translational and Functional Genomics Branch, National Human Genome Research Institute, National Institutes of Health, Bethesda, Maryland, USA; 5 Dermatology Branch, National Institute of Arthritis and Musculoskeletal and Skin Diseases, National Institutes of Health, Bethesda, Maryland, USA; 6 Division of Pediatric Infectious Diseases, Duke University School of Medicine, Durham, North Carolina, USA; 7 Division of Infectious Diseases, Texas Children’s Hospital, Department of Pediatrics, Baylor College of Medicine, Houston, Texas, USA; Argonne National Laboratory, Lemont, Illinois, USA

**Keywords:** nasal microbiota, phylogenetics, pangenomics, metabolism, *Corynebacterium accolens*, *Corynebacterium propinquum*, *Corynebacterium pseudodiphtheriticum*, *Corynebacterium tuberculostearicum*, *Corynebacterium*

## Abstract

*Corynebact*e*rium* species are globally ubiquitous in human nasal microbiota across the lifespan. Moreover, nasal microbiota profiles typified by higher relative abundances of *Corynebacterium* are often positively associated with health. Among the most common human nasal *Corynebacterium* species are *C. propinquum*, *C. pseudodiphtheriticum, C. accolens*, and *C. tuberculostearicum*. To gain insight into the functions of these four species, we identified genomic, phylogenomic, and pangenomic properties and estimated the metabolic capabilities of 87 distinct human nasal *Corynebacterium* strain genomes: 31 from Botswana and 56 from the United States. *C. pseudodiphtheriticum* had geographically distinct clades consistent with localized strain circulation, whereas some strains from the other species had wide geographic distribution spanning Africa and North America. All species had similar genomic and pangenomic structures. Gene clusters assigned to all COG metabolic categories were overrepresented in the persistent versus accessory genome of each species indicating limited strain-level variability in metabolic capacity. Based on prevalence data, at least two *Corynebacterium* species likely coexist in the nasal microbiota of 82% of adults. So, it was surprising that core metabolic capabilities were highly conserved among the four species indicating limited species-level metabolic variation. Strikingly, strains in the U.S. clade of *C. pseudodiphtheriticum* lacked genes for assimilatory sulfate reduction present in most of the strains in the Botswana clade and in the other studied species, indicating a recent, geographically related loss of assimilatory sulfate reduction. Overall, the minimal species and strain variability in metabolic capacity implies coexisting strains might have limited ability to occupy distinct metabolic niches.

**IMPORTANCE:**

Pangenomic analysis with estimation of functional capabilities facilitates our understanding of the full biologic diversity of bacterial species. We performed systematic genomic, phylogenomic, and pangenomic analyses with qualitative estimation of the metabolic capabilities of four common human nasal *Corynebacterium* species, along with focused experimental validations, generating a foundational resource. The prevalence of each species in human nasal microbiota is consistent with the common coexistence of at least two species. We identified a notably high level of metabolic conservation within and among species indicating limited options for species to occupy distinct metabolic niches, highlighting the importance of investigating interactions among nasal *Corynebacterium* species. Comparing strains from two continents, *C. pseudodiphtheriticum* had restricted geographic strain distribution characterized by an evolutionarily recent loss of assimilatory sulfate reduction in U.S. strains. Our findings contribute to understanding the functions of *Corynebacterium* within human nasal microbiota and to evaluating their potential for future use as biotherapeutics.

## INTRODUCTION

Nasal *Corynebacterium* species are frequently associated with health in compositional studies of human nasal microbiota. *Corynebacterium* are gram-positive bacteria in the phylum Actinobacteria (Actinomycetota). Based on studies from five continents, *Corynebacterium* species begin colonizing the human nasal passages before 2 months of age (1–13). *Corynebacterium* colonize both the skin-coated surface of the nasal vestibule (ak.a. nostrils/nares) and the mucus-producing nasal respiratory epithelium coating the nasal passages posterior of the limen nasi through the nasopharynx (14–19). The bacterial microbiota of the human nasal passages from the nostrils through the nasopharynx is highly similar, and we refer to it herein as the human nasal microbiota.

Pediatric nasal microbiota profiles characterized by a high relative abundance of *Corynebacterium* are often associated with health rather than a specific disease or disease-risk state in children (1, 2, 4, 7, 11–13, 20–30). In young children, the genus *Corynebacterium* (alone or with the genus *Dolosigranulum*) is negatively associated with *Streptococcus pneumoniae* nasal colonization, which is important because *S. pneumoniae* colonization is a necessary precursor to invasive pneumococcal disease (13, 20, 22, 25, 28). For example, in young children in Botswana, the genus *Corynebacterium* is negatively associated with *S. pneumoniae* colonization both in a cross-sectional study of children younger than 2 years (25) and in a longitudinal study of infants followed from birth to 1 year of age (13). In contrast to these genus-level associations, little is known about species-level prevalence and relative abundance of nasal *Corynebacterium* in children. However, in a cultivation-based study, *C. pseudodiphtheriticum* is positively associated with ear and nasal health in young Indigenous Australian children (age 2–7 years), as is *D. pigrum* (29).

In adult nasal microbiota, the prevalence of the genus *Corynebacterium* is as high as 98.6%, with highly prevalent species including *C. accolens* (prevalence of 82%), *C. tuberculostearicum* (93%), *C. propinquum* (18%), and *C. pseudodiphtheriticum* (20%), based on 16S rRNA V1-V3 sequences (Tables S4Aand B and S7 in reference 31). In these data, 82% of the adult nostril samples contained ≥2 of these 4 *Corynebacterium* species, 30% contained ≥3, and 2.4% contained all 4 species. Thus, there is a high probability of coexistence of these *Corynebacterium* species in nasal microbial communities. Like children, some adults have nasal microbiota profiles characterized by a high relative abundance of *Corynebacterium* (18, 32). At least 23 validly published species of *Corynebacterium* can be cultivated from the adult nasal passages (17, 33). However, among these, *C. accolens* and *C. tuberculostearicum* (and/or other members of the *C. tuberculostearicum* species complex [34]) followed by *C. propinquum* and *C. pseudodiphtheriticum* are the most common in human nasal microbiota, in terms of both prevalence and relative abundance (15, 17, 31, 33). Indeed, the human nasal passages appear to be a primary habitat for *C. accolens, C. propinquum*, and *C. pseudodiphtheriticum,* whereas *C. tuberculostearicum* is also prevalent, often at high relative abundances, at other human skin sites (31, 34–37).

Nasal *Corynebacterium* species interact with other common commensal/mutualistic nasal microbionts (38, 39) and with nasal pathobionts (22, 40–42). Some studies of healthy adult nasal microbiota report a negative association between *Staphylococcus aureus* and either the genus *Corynebacterium* or specific species of *Corynebacterium* (14, 31, 32, 43–47), although others do not (which might reflect strain-level variation and/or differences in populations studied). Furthermore, several small human studies support the potential use of *Corynebacterium* species to inhibit or eradicate pathobiont colonization of the human nasal passages (43, 48). This is of particular interest for *S. aureus* in the absence of an effective vaccine since *S. aureus* nasal colonization increases the risk of invasive infection at distant body sites and the infection isolate matches the colonizing isolate in ~80% of cases (49–52). Studies in mouse models further support the potential benefits of nasal *Corynebacterium* for the prevention/treatment of respiratory syncytial virus and pneumococcal infections (53, 54). Inhibition of *S. pneumoniae* or *S. aureus in vitro* by nasal *Corynebacterium* species displays strain-level variation, highlighting the importance of sequencing the genomes of multiple strains per species (13, 14, 28, 55). An increasing number of these inhibitory interactions are characterized (22, 40, 41, 54).

Some interactions between *Corynebacterium* species and other nasal microbionts are related to metabolism/metabolites. For example, *C. accolens* strains secrete the triacylglycerol lipase LipS1 to hydrolyze host-surface triacylglycerols releasing nutritionally required free fatty acids that also inhibit *S. pneumoniae in vitro* (22, 54). In addition, a positive metabolic interaction, such as cross-feeding, might be how *C. accolens*, *C. pseudodiphtheriticum,* and *C. propinquum* enhance the growth yield of the candidate mutualist *D. pigrum in vitro* (38). These examples highlight the importance of sequencing the genomes of multiple strains of each species to elucidate their metabolic capabilities and the variation that might influence interspecies interactions and contribute to promoting health-associated nasal microbiome compositions.

Overall, their ubiquity, frequent positive associations with health, and potential therapeutic use raise fundamental questions about the role of *Corynebacterium* species in human nasal microbiota. To increase genomic and metabolic knowledge of these, we performed systematic phylogenomic and pangenomic analyses of four common human nasal-associated *Corynebacterium* species. To increase the generalizability of our findings, we analyzed genomes of 87 nasal strains collected across two continents from both children and adults. Nasal strains of *C. pseudodiphtheriticum* overwhelmingly partitioned into clades by country of origin, consistent with geographically restricted strain circulation. Comparison of the core versus accessory genome of each of these four *Corynebacterium* species demonstrated that all COG categories associated with metabolism were enriched in the core genome, indicating limited strain-level metabolic variation within each species. To provide a broader context, we compare the predicted metabolic abilities of nasal *Corynebacterium* species to two well-studied *Corynebacterium* species (*C. diphtheriae* and *C. glutamicum*) and to common nasal species from other bacterial genera. Metabolic estimation revealed that these four species share the majority of KEGG modules with few species-specific, or even clade-specific, metabolic abilities. However, we found that the clade of *C. pseudodiphtheriticum* dominated by strains from the United States lacked the module for assimilatory sulfate reduction, which is key for the biosynthesis of sulfur-containing amino acids, and a representative U.S. strain was unable to grow under conditions requiring sulfate assimilation. We also validated the predictions that *C. tuberculostearicum,* alone of the four nasal species, accumulated intracellular glycogen and that *C. tuberculostearicum* and *C. accolens* generated all 20 amino acids since both grew in their absence.

## RESULTS

### *Corynebacterium pseudodiphtheriticum* displays geographically restricted strain circulation

To compare the genomic content and phylogenomic relationships among and within four *Corynebacterium* species commonly found in human nasal microbiota, we isolated strains from the nasal vestibule (nostrils) of generally healthy children and adults in the United States and from nasopharyngeal swabs of mother-infant pairs in Botswana. We compared 87 distinct nasal strain genomes to publicly available genomes of the type strain plus several other reference strains of each species, for a total of 20 reference genomes (39, 56–60) (Table S1A).

To confidently assign each new nasal isolate to a species, we first generated a maximum-likelihood phylogenomic tree based on the 632 single-copy core gene clusters (GCs) shared by the 107 strain genomes (Fig. S1A) and determined that each new nasal isolate was in a clade with the type strain of one of the nasal species (Fig. S1B; type strains in bold). *C. macginleyi* is the closest relative of *C. accolens* and these two species are challenging to distinguish by partial 16S rRNA gene sequences. Therefore, we included three *C. macginleyi* genomes in this phylogenomic analysis to confidently assign candidate *C. accolens* strains to a species. This five-species phylogenomic tree contained two major clades (Fig. S1B) confirming that *C. propinquum* and *C. pseudodiphtheriticum* are more closely related to each other, whereas *C. macginleyi*, *C. accolens*, and *C. tuberculostearicum* are more closely related to each other, with *C. macginleyi* closest to, yet distinct from, *C. accolens*. Furthermore, these two major clades are distinctly separate from each other in a broader phylogenomic representation of the genus *Corynebacterium* (Fig. S1C; Table S1C). Next, we confirmed that each strain had a pairwise average nucleotide identity (ANI) of ≥95% for core GCs compared to the type strain of its assigned species (Fig. S2A). For each species, the pairwise ANIs for core GCs were very similar to those for all shared CoDing Sequences (CDS) (Fig. S2B).

**Fig 1:**
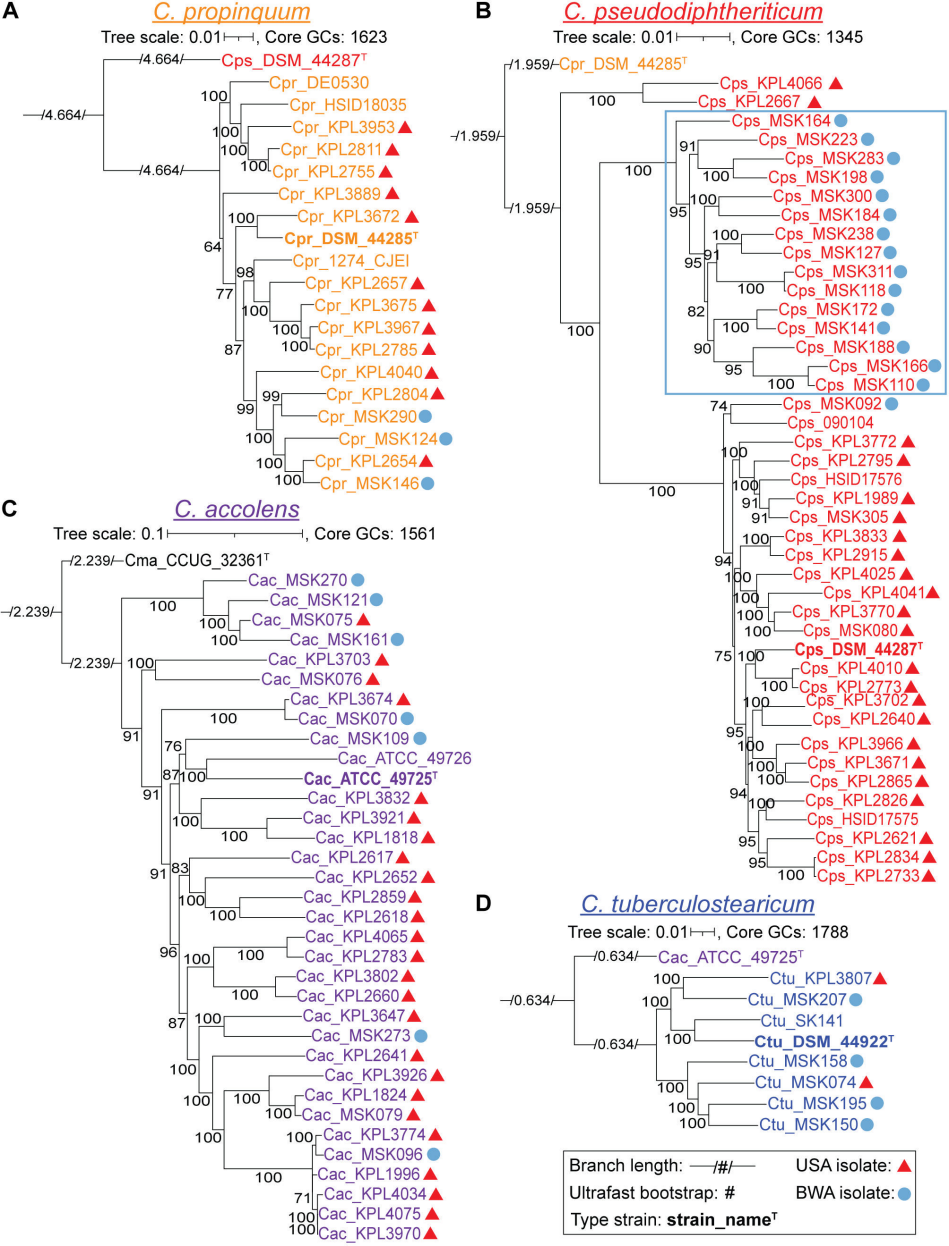
Species-specific phylogenomic trees show a geographic pattern of clades for *C. pseudodiphtheriticum*. Each panel shows a core-genome-based maximum-likelihood species-specific phylogeny. The majority (86%) of the MSK-named strains are from Botswana (blue circles), whereas all KPL-named strains are from the United States (red triangles). (**A**) Phylogeny of 19 *C*. *propinquum* strains based on 1,623 core GCs shows two major clades (BIC value 9762417.2123). (**B**) Phylogeny of 43 *C*. *pseudodiphtheriticum* strains based on 1,345 core GCs shows three major clades, one of which is entirely composed of strains from Botswana (15/15, outlined in light blue), whereas the other two have a majority of the U.S. nasal strains (red triangles), with 2/2 and 20/22, respectively (BIC value 10177769.6675). The branching pattern separating the Botswana and U.S. clades was well supported with ultrafast bootstrap values ≥ 95%. (**C**) Phylogeny of 34 *C*. *accolens* strains based on 1,561 core GCs with the majority collected in the United States shows most Botswana strains dispersed throughout (BIC value 10700765.2332). (**D**) Phylogeny of eight *C. tuberculostearicum* strains based on 1,788 core GCs with 6 nasal isolates from Botswana and the United States (BIC value 10452720.3067). For each species-specific phylogeny, the type strain from the most closely related species (Fig. S1B) serves as the outgroup. Each phylogeny was made from all shared conservative core GCs for a given species (Fig. S2C), including the subset of GCs that were absent in the corresponding outgroup (Fig. S2D), to provide the highest possible resolution among the strains within each species. A large majority of the branches have highly supported ultrafast bootstrap values with the lowest at 64 on an ancestral branch in the *C. propinquum* phylogeny. Type strains are indicated in bold with a superscript T. Ancestral branch lengths are indicated numerically within a visually shortened branch to fit on the page. Phylogenies were generated with IQ-Tree v2.1.3 using the model finder, edge-linked-proportional partition model, and 1,000 ultrafast rapid bootstraps.

To assess the evolutionary relationships between nasal isolates from both the United States and Botswana, we produced individual maximum-likelihood phylogenomic trees for each species (Fig. 1) based on its conservative core genome (Fig. S2C). These species-specific phylogenies provided a refined view of the relationships between strains based on the larger number of shared single-copy core GCs within each species (ranging from 1,345 to 1,788). To better approximate the root of each species-specific tree (Fig. 1), we used the type strain of the most closely related species in the multispecies phylogenomic trees (Fig. S1) as the outgroup (Fig. S2D). With a relatively even representation of Botswana and U.S. strains (40% vs. 58%), the phylogenomic tree for *C. pseudodiphtheriticum* had two large, well-supported clades dominated, respectively, by nasal strains from Botswana (15/15) or from the United States (20/22), indicating a restricted distribution of strains by country (Fig. 1B). We avoided calculating geographic proportions within major clades for *C. propinquum* (Fig. 1A) and *C. accolens* (Fig. 1C) because of the disproportionately high representation of U.S. strains (80%) and for *C. tuberculostearicum* (Fig. 1D) because there were only six nasal strains, five of which were from Botswana. Within these limitations, the phylogenomic analysis of these three species revealed some remarkably similar strains present in samples collected in the United States and Botswana based on their residing together in terminal clades. This raises the possibility that a subset of strains from each of these three species might have a wide geographic distribution, spanning from at least Botswana to Massachusetts.

### The sizes of the core genomes of four common nasal *Corynebacterium* species have leveled off

**Fig 2:**
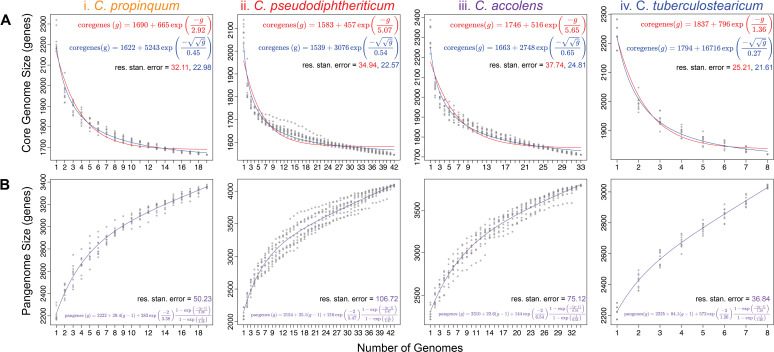
The four nasal *Corynebacterium* species have core genomes that have leveled off and pangenomes that remain open. (A) All four species have a core genome that has leveled off using a Tettelin curve fit model. (Ai) The *C. propinquum* core genome (*n* = 19) leveled off at ~12 genomes. (Aii) The *C. pseudodiphtheriticum* core genome (*n* = 42) leveled off at ~19 genomes. (Aiii) The *C. accolens* core genome (*n* = 33) leveled off at ~21 genomes. (Aiv) The *C. tuberculostearicum* core genome (*n* = 8) leveled off at ~7 genomes. Two best-fit curve line models are shown for the core genome: Tettelin (red) and Willenbrock (blue). (**B**) The pangenomes for the four *Corynebacterium* species (**i–vi**) remain open as indicated by the continuous steep slope of the best-fit line shown in purple. Core and pangenome size estimations were calculated from 10 random genome samplings (represented by gray dots) using the OMCL algorithm predicted GCs with GET_HOMOLOGUES v24082022.

Based on rarefaction analysis, the core genomes of *C. propinquum, C. pseudodiphtheriticum, C. accolens,* and *C. tuberculostearicum* have reached a stable size and are unlikely to decrease much further with the sequencing of additional strains (Fig. 2A). Based on the respective Tettelin curves (red line), the *C. tuberculostearicum* core genome stabilized first at ~7 genomes; however, with the fewest genomes at 8, this might be an upper bound that will continue to decrease with additional strain genomes. In comparison, *C. pseudodiphtheriticum* had the largest number of strain genomes at 43, with pairwise ANIs of ≥96.2% (Fig. S2Aii), and its core genome stabilized last at ~37 genomes.

The proportion of an individual genome of each of these nasal *Corynebacterium* species devoted to conservative core GCs ranged from 72% for *C. pseudodiphtheriticum* (1517/2105) to 79% for *C. tuberculostearicum* (1788/2250). This is based on the average number of CDS per genome (Table 1). The average/median genome size for each species ranged from ~2.33/2.33 Mb for *C. pseudodiphtheriticum* to ~2.51/2.52 Mb for *C. propinquum* with the average/median predicted CDS per genome ranging from 2,105/2,096 for *C. pseudodiphtheriticum* to 2,265/2,272 for *C. propinquum* (Table 1). These sizes and proportions are consistent with the reduced genome size and GCs per genome of host-associated compared to environment-associated *Corynebacterium* species. For example, Swaney et al. report that environmental *Corynebacterium* species have a larger median genome size of 3.03 Mb and more GCs per genome (with an average of 2,664) compared to host-associated *Corynebacterium* species (61).

**TABLE 1:** Basic genomic information for four common human nasal-associated *Corynebacterium* species

*Corynebacterium* species	No. of strain genomes (no. of nasal isolates)	Average (median) genome size (Mb)	Average (median) CDS/genome	Average (median) G+C%	Conservative core GCs/species^*a*^	Pangenome GCs/species^*b*^	Country (no. USA/no. BWA	Age range (years) of strain donors
*C. propinquum*	19 (15)	2.51 (2.52)	2,265 (2,272)	56.47 (56.48)	1,623	3,777	12/3	0.3–72
*C. pseudodiphtheriticum^c^*	43 (39)	2.33 (2.33)	2,105 (2,096)	55.29 (55.29)	1,517	4,590^*c*^	22/17	0.2–55
*C. accolens^d^*	34 (32)	2.50 (2.49)	2,304 (2,294)	59.46 (59.44)	1,679	4,220^*d*^	25/7	0.1–62
*C. tuberculostearicum*	8 (6)	2.39 (2.39)	2,250 (2,253)	59.86 (59.88)	1,788	3,232	2/4	0.1–39

^*a*^ GET_HOMOLOGUES conservative core GCs predicted from the consensus of BDBH, OMCL, and COGS algorithms.

^*b*^ GET_HOMOLOGUES pangenome GCs predicted from the consensus of OMCL, and COGS algorithms.

^*c*^ Cps_090104 was removed from this analysis (see Materials and Methods).

^*d*^ Cac_ATCC_49756 was removed from this analysis (see Materials and Methods).

### The pangenomes of these four human nasal-associated *Corynebacterium* species remain open

With the number of strain genomes analyzed (Table 1), the pangenome of each of the four species continued to increase with each additional new genome, indicating that all are open (Fig. 2B). Parameters used to generate a pangenome via rarefaction yielded an overly conservative estimate of its size in GCs. Therefore, we used two other approaches to estimate the number of GCs in the pangenome for each species. These pangenome composition estimates are a lower bound for each species and will increase with the sequencing of additional strain genomes. Starting with GET_HOMOLOGUES, we estimated pangenome size using the COG triangle and OMCL clustering algorithms (Fig. S3A). The pangenome size and its proportion contributed by core versus accessory GCs for each species ranged from 3232 GCs with 56% core and 44% accessory for *C. tuberculostearicum* to 4590 GCs with 33% core and 67% accessory for *C. pseudodiphtheriticum* (Table 2). The 56% core percentage for *C. tuberculostearicum* is likely an overestimate since this pangenome is based on only eight genomes. This range of 33% to 56% for core genes per pangenome is similar to estimates for other human upper respiratory tract microbionts, such as *D. pigrum* (31%) (62), *S. aureus* (36%), and *Streptococcus pyogenes* (37%) (63).

**TABLE 2:** Pangenomic estimation of human nasal-associated *Corynebacterium* species based on three different platforms

Platform species	Pangenome size (GCs)	% core GCs/pangenome	% accessory GCs/pangenome
GET_HOMOLOGUES^*a*^			
*C. propinquum*	3,777	43	57
*C. pseudodiphtheriticum*^*b*^	4,590	33	67
*C. accolens*^*c*^	4,220	40	60
*C. tuberculostearicum*	3,232	56	44
Anvi’o			
*C. propinquum*	3,108	59	40
*C. pseudodiphtheriticum*	3,590	48	51
*C. accolens*	3,427	57	42
*C. tuberculostearicum*	2,907	66	32
PPanGGOLiN		% persistent	
*C. propinquum*	Est.^*d*^	63	37
*C. pseudodiphtheriticum*	Est.	49	51
*C. accolens*	Est.	59	41
*C. tuberculostearicum*	Est.	69	31

^*a*^ GET_HOMOLOGUES pangenome size, % core, and % accessory are from the consensus of OMCL and COGS algorithms.

^*b*^ Cps_090104 was removed from this analysis (see Methods).

^*c*^ Cac_ATCC_49756 was removed from this analysis (see Methods).

^*d*^ Est., pangenome size was estimated in anvi’o then the GCs imported into PPanGGOLiN to estimate persistent vs accessory genome percentages.

Next, we used anvi’o to estimate the core- and pangenomes (64). The number of GCs in the core genome of each species estimated with GET_HOMOLOGUES was within 6%–13% of those estimated with anvi’o; however, the GET_HOMOLOGUES estimated pangenome sizes were 11%–24% larger (Fig. S3A; File S1). Consistent with this, the estimated single-copy core as a proportion of the pangenome using anvi’o ranged from 41% to 64% (Fig. S3B).

We also used anvi’o to visualize the strain-level variation in gene presence and absence within the four human nasal-associated *Corynebacterium* species (Fig. S3B). Manually arraying the genomes in anvi’o to correspond with their species-specific phylogenomic tree (Fig. 1) showed that some blocks of gene presence/absence correlated with the core-genome-based phylogenetic relationships among strains, but others did not (Fig. S3B). This is consistent with gene gain and loss playing a role in strain diversification with some of this due to mobile genetic elements and horizontal gene transfer (65, 66).

### Gene clusters assigned to the COG categories associated with metabolism are highly enriched in the core genomes of common nasal *Corynebacterium* species

**Fig 3:**
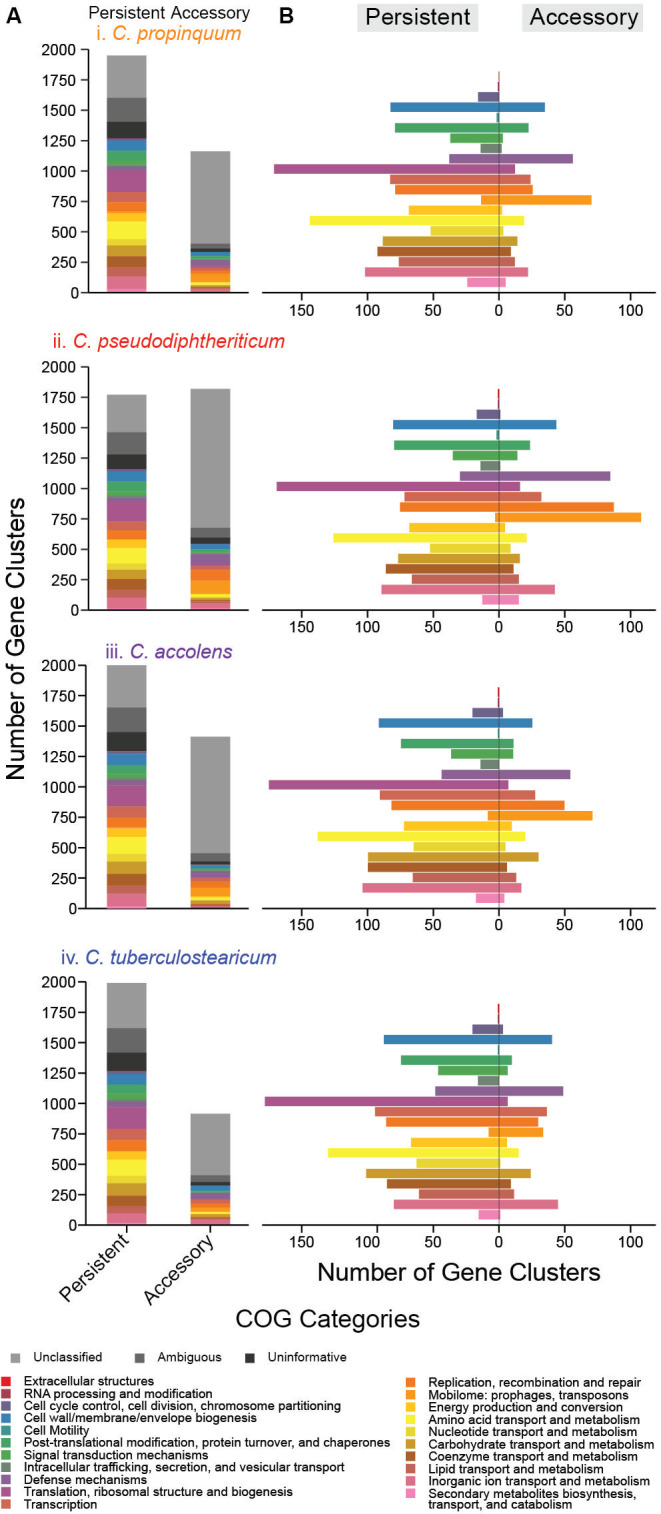
GCs assigned to COG metabolism categories are overrepresented in the persistent compared to the accessory genomes of each species indicating limited strain-specific metabolism. We identified the COG functional annotations for GCs using anvi’o and then used PPanGGOLiN to assign GCs to the persistent vs accessory genome. (**A**) Over one-third of the GCs in each species (**i–iv**) were assigned as uninformative (black), ambiguous (dark gray), or unclassified (gray) across both the persistent and accessory genomes. The combined percentage of each of these categories out of all the genes per species was 38.1% *Cpr*, 37.9% *Cps*, 37.1% *Cac*, and 38.3% *Ctu*. For each species, the percentage of GCs with an informative COG assignment was higher in the persistent genome, 64.9% *Cpr* (1262), 65.3% *Cps* (1156), 64.7% *Cac* (1300), and 63.5% *Ctu* (1264), than in the accessory genome, with 28.9% *Cpr* (336), 29.9% *Cps* (543), 25.7% *Cac* (363), and 35.6% Ctu (326). (**B**) Functional enrichment of GCs in the persistent vs the accessory genome for the different COG categories. Metabolic COG categories, for example, those involved in energy production (pale orange), or amino acid (yellow), nucleotide (gold), carbohydrate (khaki), and lipid metabolism (dark salmon), were enriched in the persistent genome of each species. By contrast, mobilome (bright orange) and to a lesser extent defense mechanisms were enriched in the accessory genomes. Each *Corynebacterium* species shared similar COG functional enrichment ratios of GCs in its persistent vs its accessory genome.

To predict and compare functions based on the pangenomes of each species, we assigned GCs to COG categories and used PPanGGOLiN to define the persistent versus the accessory genome (Table S2) (67). We used the PPanGGOLiN estimation of the persistent genome rather than the traditionally defined core genome for this analysis because PPanGGOLin limits the effect of technical artifacts and/or strain-level gene loss events on the assessment of persistently shared gene clusters by considering the genomic context via examining genetic contiguity. As is common in bacteria, only about 63%–65% of the GCs in the persistent genome and 26%–36% of the GCs in the accessory genome of each species had an informative assignment to a definitive COG category (Fig. 3A; Fig. S4A). There was also variability in the size of the accessory genome among strains within each species (Fig. S4A and B). We next generated functional enrichment plots for COG categories in the persistent versus the accessory genome of each species (Fig. 3B). GCs assigned to “Mobilome: prophages, transposons” (mobile genetic elements [MGEs]; orange bar in Fig. 3B) were overrepresented in the accessory genome of each species with the ratio of GCs in the accessory/persistent genome ranging from 4.2 (*C. tuberculostearicum*) to 36.1 (*C. pseudodiphtheriticum*). GCs assigned to “defense mechanisms” (purple bar in Fig. 3B), which protect bacteria from MGEs, were more evenly distributed with the ratio of GCs in the accessory/persistent genome ranging from 1 (*C. tuberculostearicum*) to 2.9 (*C. pseudodiphtheriticum*). These findings are consistent with pangenomic analyses of other bacterial species, including our prior analysis of the candidate beneficial nasal bacterium *D. pigrum* (62).

Our COG-enrichment analysis also showed that all the COG categories associated with metabolism, from “energy production and conversion” (pale orange) through “secondary metabolites biosynthesis, transport, and catabolism” (pink) in Fig. 3B, were highly overrepresented in the persistent (or core) genome of each species with ratios of accessory/persistent ranging from 0.02 to 0.56 (median of 0.16). The exception was an accessory/persistent GC ratio of 1.2 for “secondary metabolites biosynthesis, transport, and catabolism” in *C. pseudodiphtheriticum*. The overrepresentation of metabolic categories in the persistent genome of each species points to limited strain-level variation in metabolic capabilities, such as carbohydrate or amino acid metabolism. This contrasts with our previous analysis of *D. pigrum* in which GCs assigned to the COG category “carbohydrate transport and metabolism” are enriched in the accessory genome (ratio 1.66) (62).

### Common human nasal-associated *Corynebacterium* species have a largely shared metabolic capacity

**Fig 4:**
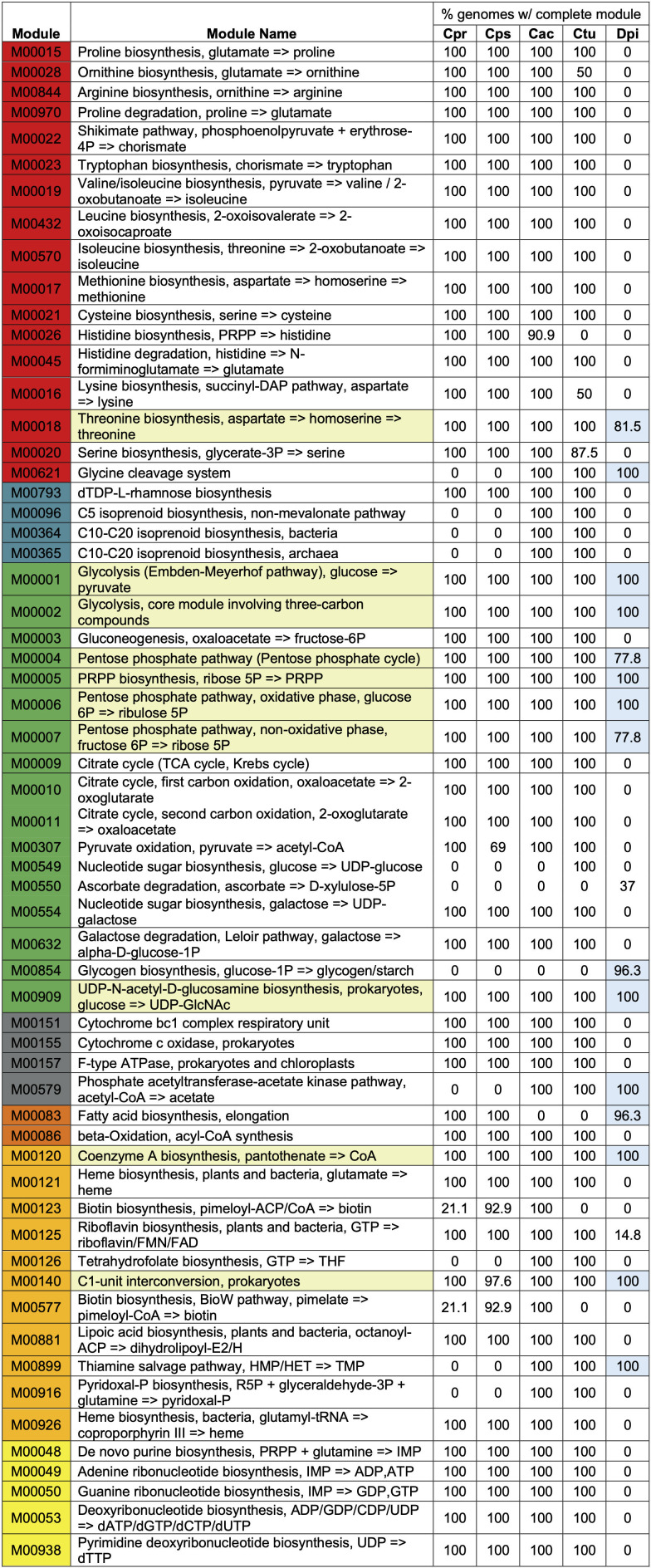
List of estimated complete KEGG modules. Module ID numbers are colored based on their KEGG module subcategory; red: “amino acid metabolism,” dark teal: “biosynthesis of terpenoids and polyketides,” green: “carbohydrate metabolism,” gray: “energy metabolism,” dark orange: “lipid metabolism,” orange: “metabolism of cofactors and vitamins,” yellow: “nucleotide metabolism.” Complete KEGG modules shared by *D. pigrum* and all four *Corynebacterium* species are highlighted in pale yellow. Complete KEGG modules shared by the majority (≥78%) of the 27 *D. pigrum* strain genomes are highlighted in light blue. PRPP is 5-phosphoribosyl diphosphate, DAP is diaminopimelate, FMN is flavin mononucleotide, FAD is flavin adenine dinucleotide, ACP is acyl-carrier protein, HMP is 4-amino-5-hydroxymethyl-2-methylpyrimidine, HET is 5-(2-hydroxyethyl)-4-methylthiazole, and TMP is thiamine monophosphate.

**Fig 5:**
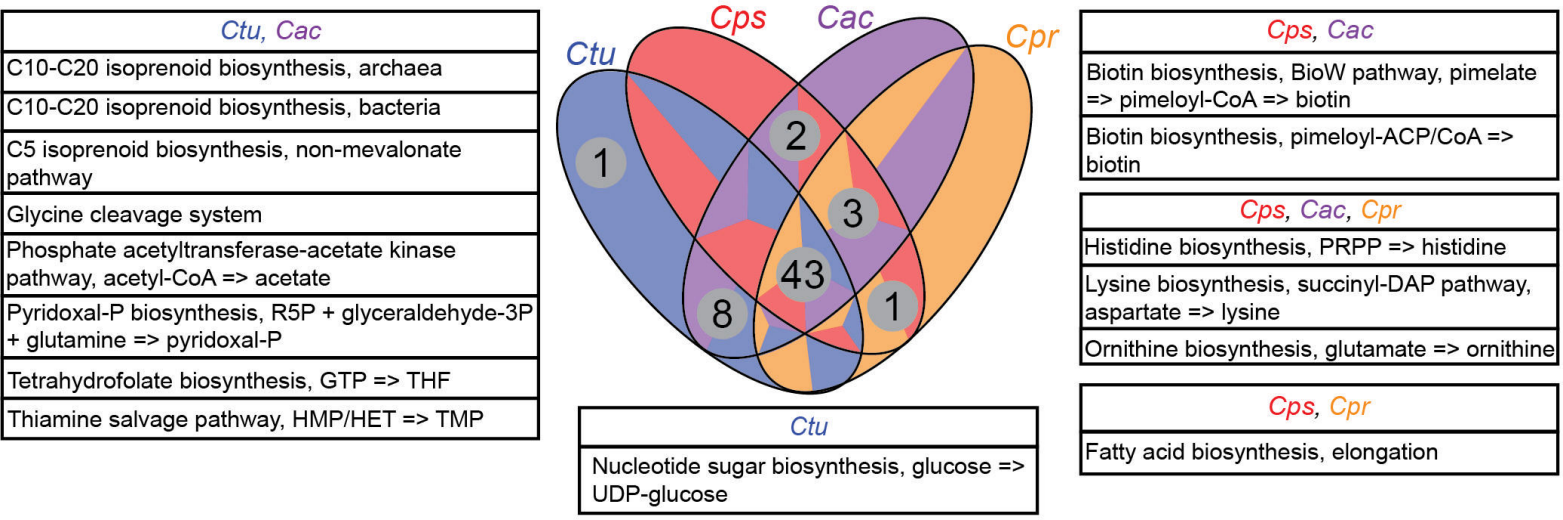
These four common human-nasal-associated *Corynebacterium* species have a largely shared metabolic capacity. The Venn diagram summarizes the results of an enrichment analysis of complete KEGG modules (stepwise completion score = 1) by species using anvi’o. Modules that both had an adjusted *q*-value >1e−9 and were complete in at least 87% of the analyzed genomes were categorized as shared between the four *Corynebacterium*. Modules with an adjusted *q*-value <1e−9 were considered enriched in their associated group of ≤3 species and are shown in boxes surrounding the Venn diagram. Species labels: *C. propinquum* (*Cpr*)*, C. pseudodiphtheriticum (Cps), C. accolens (Cac),* and *C. tuberculostearicum (Ctu*).

Based on 16S rRNA V1-V3 sequences, 82% of adults are colonized with ≥2 of these 4 *Corynebacterium* species (see Tables S4Aand B, and S7 in reference 31). This, combined with the enrichment of GCs assigned to metabolism COG categories in each persistent genome, led us to hypothesize that there would be much species-specific variation in core metabolic capabilities enabling the different nasal *Corynebacterium* species to occupy distinct metabolic niches within human nasal microbiota. To test our hypothesis, we used anvi’o to assign genes to Kyoto Encyclopedia of Genes and Genomes (KEGG) Orthology family (KO) annotations (Table S3A) and to estimate KEGG module completeness (Table S4A and B). In contrast to our hypothesis, we learned that *C. propinquum*, *C. pseudodiphtheriticum*, *C. accolens,* and *C. tuberculostearicum* encode highly conserved core metabolic capabilities sharing 43 of 58 (74%) detected fully complete KEGG modules (Fig. 4 and 5). Next, we identified modules enriched in one or more of the four species, with various combinations of three of the four species sharing eleven additional modules (19%) and combinations of two of the four species sharing three additional modules (Table S4D). There were a few differences between the *C. propinquum-C. pseudodiphtheriticum* clade and the *C. accolens-C. tuberculostearicum* clade (Fig. S1B), with one and eight clade-specific KEGG modules, respectively. Only *C. tuberculostearicum*, which is broadly distributed across human skin sites as well as in the nasal passages (31, 34–37), was predicted to encode one complete KEGG module that was absent in the other three nasal species, as discussed in detail later.

### Nasal *Corynebacterium* species encode for central carbohydrate metabolism

To contextualize our findings within the genus *Corynebacterium*, we included in our anvi’o analyses the genomes of the type strains of two well-studied *Corynebacterium* species: *C. glutamicum* ATCC 13032 (*C. glutamicum*^T^) (68) and *C. diphtheriae* NCTC11397 (*C. diphtheriae*^T^) (Fig. S1; S5). The metabolism of the soil bacterium *C. glutamicum* is the best studied of *Corynebacterium* species (69). *C. diphtheriae* colonizes the human pharynx and toxigenic strains cause the human disease diphtheria (70). Along with *C. diphtheriae*^T^ and *C. glutamicum*^T^, the four human nasal *Corynebacterium* species all encoded complete modules for glycolysis and gluconeogenesis, the pentose phosphate pathway and phosphoribosyl pyrophosphate, and the tricarboxylic acid (TCA) cycle as part of central carbon metabolism (Fig. 4). In terms of other carbohydrate metabolism, all four nasal species, plus *C. diphtheriae*^T^ and *C. glutamicum*^T^, also encoded a complete module for UDP-N-acetyl-D-glucosamine biosynthesis (M00909), a precursor of cell wall peptidoglycan (71). All four species plus *C. diphtheriae*^T^, but not *C. glutamicum*^T^, also encoded the module for the Leloir pathway for galactose degradation (M00632).

### Nasal *Corynebacterium* species encode for the synthesis of key biosynthetic cofactors, vitamins, and electron transport chain components

The strain genomes of all four species contained complete modules for biosynthesis of several cofactors and vitamins required for synthesis of essential biomolecules and central metabolism (Fig. 4). These include coenzyme A (M00120), required for the TCA cycle; lipoic acid (M00881), an organosulfur cofactor required in central metabolism (72); and C1-unit interconversion (M00140), which is connected to biosynthesis of tetrahydrofolate. Consistent with an intact TCA cycle, all four also had complete modules for the biosynthesis of key compounds involved in the electron transport chain, including heme (M00121, M00926), and riboflavin (M00125). *C. accolens* and *C. tuberculostearicum* also encoded biosynthesis of pyridoxal 5′-phosphate (M00916), which is a coenzyme in many transamination reactions (73); and tetrahydrofolate biosynthesis (M00126), which acts as a carrier for single carbon groups (Fig. 4). We also detected the modules for coenzyme A, lipoic acid, heme, riboflavin, and pyridoxal 5′-phosphate in both *C. glutamicum*^T^ and *C. diphtheriae*^T^. Of note, modules for the biosynthesis of cobalamin/vitamin B12 (M00925, M00924, and M00122) were incomplete or absent in all four nasal *Corynebacterium* species. However, the nasal *Corynebacterium* species also encoded for the version of enzymes that are expected to be cobalamin independent, for example, *metE* rather than the B12-dependent *metH*, so are unlikely to require cobalamin, consistent with predictions from Shelton and colleagues (74).

### Nasal *Corynebacterium* species share necessary modules for nucleotide synthesis and energy generation

All four of these common nasal *Corynebacterium* species had five complete modules related to nucleotide metabolism (yellow in Fig. 4) and three complete modules involved in ATP synthesis (M00151, M00155, and M00157). Lastly, all four had complete modules for dTDP-L-rhamnose biosynthesis (M00793), a precursor to rhamnose cell wall polysaccharides. Rhamnose is part of the polysaccharide linker between peptidoglycan and arabinogalactan in members of *Mycobacteriales*, including *Corynebacterium* (71). Many of these 9 KEGG modules are also present in other common nasal microbionts with 8/9 in *Cutibacterium acnes* KPA171202 (75), 6/9 in *S. pneumoniae* TIGR4 (76) and *S. aureus* USA300_FPR3757 (77), 5/9 in *Staphylococcus epidermidis* RP62A (78), as well as 9/9 in *C. diphtheriae*^T^, and *C. glutamicum*^T^. However, all 9 of these modules were incomplete or absent across all 27 *D. pigrum* strains (62) (Table S4C).

### Nasal *Corynebacterium* species encode for biosynthesis of UDP-glucose via UDP-galactose

The UDP-glucose biosynthesis module (M00549) was fully detected in *C. tuberculostearicum* but was missing a step in the other nasal *Corynebacterium* and in *D. pigrum* (and present in all the other species analyzed [Table S4C]). UDP-glucose is a key part of central metabolism. It is the activated form of glucose that serves as a precursor for other activated carbohydrates and is used by most organisms for glucosyl transfers. Phosphoglucomutase (*pgm*) performs the second step in its three-step biosynthesis module. We identified a GC that included *pgm* from *C. glutamicum*, *C. tuberculostearicum,* and the skin-associated *Corynebacterium* species but lacked sequences from the other nasal *Corynebacterium* species. However, all four nasal *Corynebacterium* species encoded the module for UDP-galactose biosynthesis (M00554) plus a UDP-glucose 4-epimerase (K01784) to covert UDP-galactose to UDP-glucose. By contrast, the *D. pigrum* genomes encoded a *pgm* but lacked the third step, a UDP-sugar pyrophosphorylase, or a UTP--glucose-1-phosphate uridylyltransferase (*galU*), for the addition of glucose to UDP and also lacked the comparable step for the addition of galactose to UDP suggesting the genes encoding for these steps are not yet included in the KEGG orthology.

### *C. tuberculostearicum* performs glycogen metabolism, unlike the other three nasal-associated *Corynebacterium* species

Based on anvi’o KEGG module reconstruction (Table S4C), three of the nasal *Corynebacterium* species had a stepwise completeness score of zero for the modules for glycogen synthesis and degradation (M00854 and M00855), whereas *C. tuberculostearicum* had scores of 0.5 and 0.67, respectively, for these two modules. Likewise, *C. glutamicum*^T^ had completeness scores of 0.5 and 0.67 for these modules and was missing the same steps as *C. tuberculostearicum*. However, published data indicate *C. glutamicum* both synthesizes and degrades glycogen pointing to the use of glycogen for energy storage (79, 80). It encodes four genes for glycogen synthesis (*glgC*, *glgA*, *glgB,* and *glgE*) and three genes for glycogen degradation (*glgX* and two copies of *glgP*). After identifying the predicted enzymatic function (KO number) that anvi’o assigned to the product of each of these six genes, we noted that all of these KOs are enriched in *C. tuberculostearicum* compared to the other three nasal *Corynebacterium* species (Table S3B). However, the KEGG module definitions for M00854 and M00855 lack the KOs corresponding to *glgA* (K16148), *glgE* (K16147), and *glgX* (K01214) in *C. glutamicum*^T^ and *C. tuberculostearicum*, and likely other *Corynebacterium* species. In fact, when we included these three KOs in the glycogen metabolism predictions for representative strains of other *Corynebacterium* species and non-*Corynebacterium* human nasal bacterial species, we identified complete glycogen synthesis pathways in *Corynebacterium diphtheriae* NCTC11397, the three common skin *Corynebacterium* species *Corynebacterium simulans* PES1 (81)*, Corynebacterium kroppenstedtii* DSM 44395 (82), and *Corynebacterium amycolatum* FDAARGOS_1108 (83) as well as in strains of other common nasal bacteria: *C. acnes* KPA171202 (75), *S. pneumoniae* TIGR4 (76), and *D. pigrum* (26/27 strain pangenome) (62). Glycogen degradation was complete in all of those strains except *C. kroppenstedtii* and *D. pigrum*, which were missing enzymatic activities for the first or second steps, respectively, of the module.

**Fig 6:**
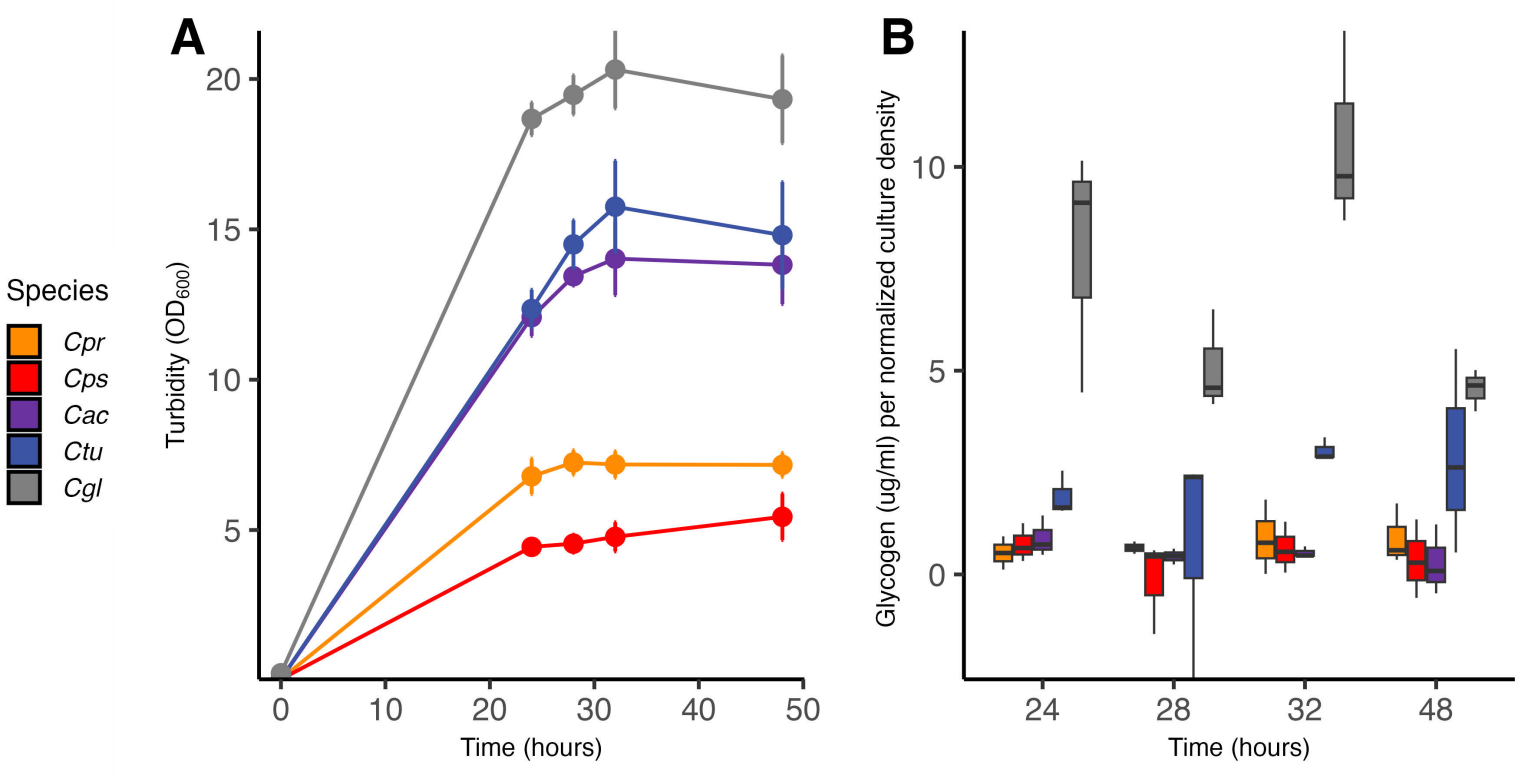
*C. tuberculostearicum* accumulates intracellular glycogen. (**A**) Growth curves for each of the nasal *Corynebacterium* species at 34°C in a MOPs-buffered CDM with all 20 amino acids and 5% glucose, with *C. glutamicum* as a positive control for the assay. *C. propinquum* and *C. pseudodiphtheriticum* reached stationary phase at a lower optical density at wavelength 600 nm (OD_600_) than did *C. tuberculostearicum* and *C. accolens*, with *C. glutamicum*^T^ reaching the highest. (**B**) Overall, *C. tuberculostearicum* accumulated more intracellular glycogen than the other three nasal species (*P*-value range 0.033 to 0.009), and less than *C. glutamicum* (*P* = 6.64 × 10^−10^). Intracellular glycogen content was quantified in µg/mL at timepoints between 24 and 48 hours. Statistics were done using a linear mixed model with species as a fixed effect and time points as a random effect. Boxplots show the median with the first and third quartiles. Data (**A and B**) are from *n* = 3 independent experiments. Species labels: *C. propinquum* KPL3953 (*Cpr*)*, C. pseudodiphtheriticum* KPL1989 (*Cps*)*, C. accolens* KPL1818 (*Cac*)*, C. tuberculostearicum* MSK074 (*Ctu*), and *C. glutamicum* DSM 20300^T^ (*Cgl*).

Based on this analysis, we predicted that *C. tuberculostearicum* can synthesize and degrade glycogen like *C. glutamicum*. To test for this, we measured intracellular concentrations of glycogen in the four nasal *Corynebacterium* species, using *C. glutamicum*^T^ as a positive control. We grew each in a liquid chemically defined medium (CDM) supplemented with all 20 amino acids and 5% glucose (Fig. 6A). We used a standardized estimated number of cells, based on OD_600_, to assay for intracellular glycogen. Under these conditions, *C. tuberculostearicum* harbored increased intracellular glycogen compared to the basal level observed in the other nasal strains (Fig. 6B). In *C. glutamicum,* glycogen both serves in carbohydrate storage and can be degraded to allow for a rapid response to hyperosmotic conditions via biosynthesis of trehalose (79, 80). In our analysis, *C. tuberculostearicum* lacked *treYZ*, which encodes the main pathway for the degradation of glycogen to trehalose in *C. glutamicum* (84, 85). However, it encoded for *treS* (K05343) and TreS functions both as an amylase, degrading glycogen to maltose, and as an isomerase, interconverting maltose and trehalose in *Mycobacterium smegmatis* (86). We speculate that since *C. tuberculostearicum* is also common on human skin (31, 34–37), where it would encounter high salt environments and increased osmotic stress, glycogen metabolism might provide it with a rapid source for producing trehalose as an osmoprotectant.

### Nasal *Corynebacterium* species can synthesize proteinogenic amino acids

Our overall analysis estimated that the vast majority of the analyzed strains of these four common human nasal *Corynebacterium* species can synthesize all 20 standard amino acids. In most of the genomes, we detected complete biosynthetic modules for 11 amino acids, including the hydrophobic amino acids (isoleucine, leucine, methionine, valine, and tryptophan); the polar uncharged amino acids (serine and threonine); the charged amino acids (arginine and lysine); cysteine; and proline (Fig. 4).

Based on anvi’o KEGG module reconstruction (Table S4C), the four nasal *Corynebacterium* species and *C. glutamicum*^T^ ATCC13032 have incomplete modules for phenylalanine (M00024) and tyrosine (M00025) due to lack of the corresponding aromatic aminotransferase KO. However, published experimental data confirm that the protein encoded by the *aroT* gene (NCgl0215 or CGL_RS01140) in *C. glutamicum*^T^ acts as an aromatic aminotransferase on substrates phenylpyruvate (O-Phe) and 4-hydroxyphenylpyruvate (O-Tyr) (87). Using anvi’o to cluster all of the genomes for the four nasal *Corynebacterium* species plus *C. glutamicum*^T^, we identified a GC that includes *C. glutamicum aroT* (annotated as K00817) and has matches in all of the analyzed strains. Based on this, we predicted that all four nasal *Corynebacterium* species should indeed be able to synthesize phenylalanine and tyrosine in the same way as *C. glutamicum*.

Substrate specificity of aminotransferase enzymes is notoriously difficult to predict from sequencing data due to the high degree of amino acid similarity within this enzyme family. Moreover, substrate promiscuity often leads to nonobservable phenotypes for deletion mutants of many aminotransferase genes (87–89). Both in *E.coli* (90) and *C. glutamicum* (87), this substrate overlap is especially significant for hydrophobic amino acids, including phenylalanine and tyrosine, which might explain why the initial anvi’o KEGG predictions were inaccurate for *aroT*. In fact, the identified *aroT* gene had an anvi’o KOfam annotation of histidinol-phosphate aminotransferase (K00817), and in *C. glutamicum*^T^ this motif is predicted in both *aroT* and *hisC* (named cg0267 and cg2304, respectively, in reference 88). In that study, *aroT* had higher similarity to a set of histidinol-phosphate aminotransferases with broader substrate specificity, whereas *hisC* was most similar to an aminotransferase specific for histidine biosynthesis. The *hisC* gene is also located in an operon with other genes involved in histidine biosynthesis. In our genomes of interest, we also identified another GC including the *C. glutamicum*^T^ *hisC*.

The majority (91/102) of the genomes had a complete KEGG module for histidine biosynthesis (M00026), except that all *C. tuberculostearicum* and 3 *C. accolens* genomes were missing K01693. However, the gene cluster analysis revealed matches to this KO, corresponding to *C. glutamicum*^T^ *hisB*, in all 11 of these genomes, leading us to predict that all of the strains of interest can biosynthesize histidine.

### The *C. pseudodiphtheriticum* phylogeny revealed a recent and geographically localized loss of assimilatory sulfate reduction

**Fig 7:**
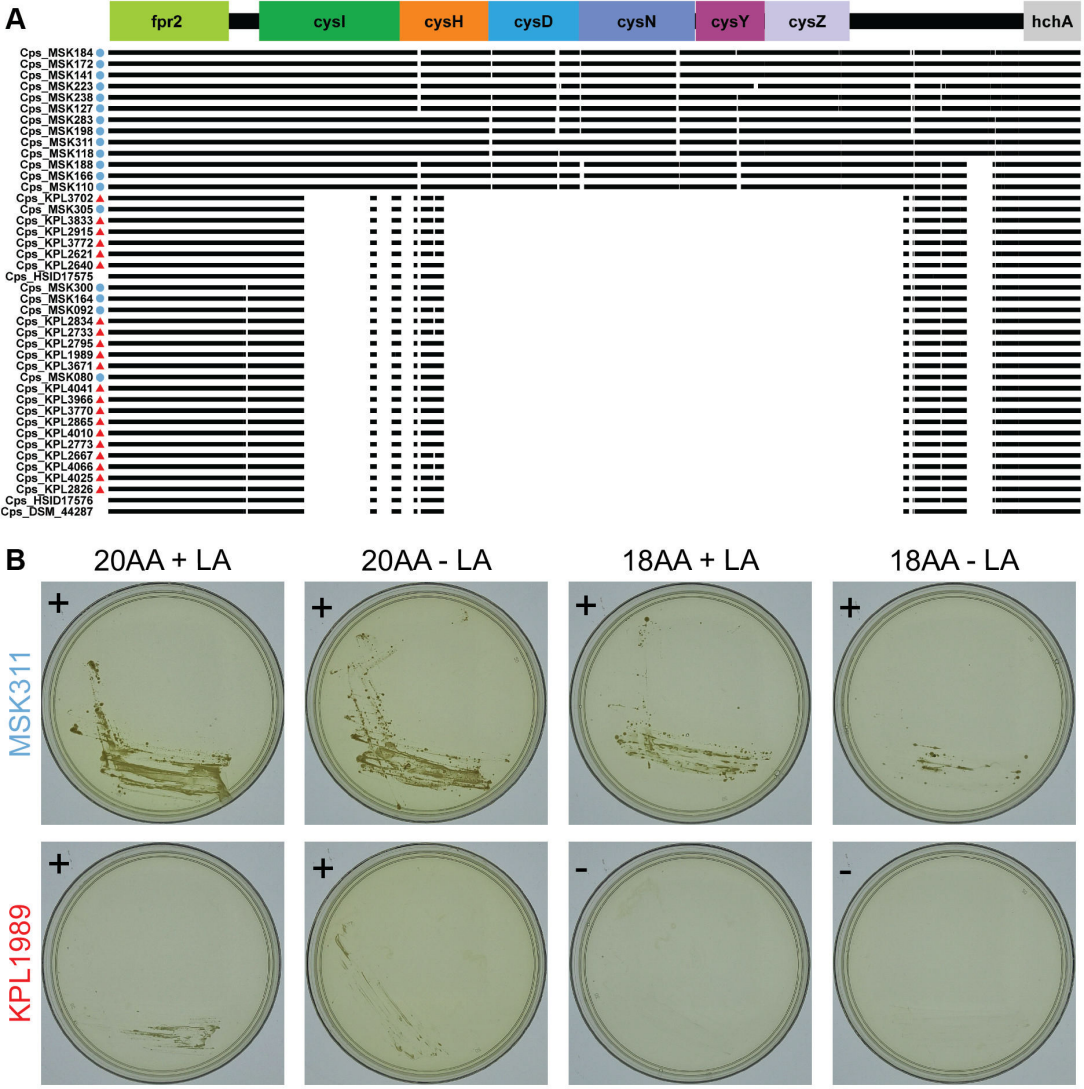
*C. pseudodiphtheriticum* strains from the Botswana clade in Fig. 1B can perform assimilatory sulfate reduction. (**A**) Most of the Botswana *C. pseudodiphtheriticum* strains encode genes with KO annotations equivalent to those in the *C. glutamicum*^T^ *fpr2-cysIXHDNYZ* operon, which is required for assimilatory sulfate reduction. The figure represents the MAFFT multiple sequence alignment with strain genomes from Botswana (blue circles) and from the United States (red triangles). (**B**) The Botswana *C. pseudodiphtheriticum* strain MSK311, which encodes the *fpr2cysIXHDNYZ* operon, grew on chemically defined agarose medium with sulfate as the only source of sulfur (KS-CDM 18AA -LA) indicating the ability to assimilate sulfate, whereas the U.S. strain KPL1989, which lacks this operon, did not. Representative strains from the Botswana (blue font) and United States (red font) clades in Fig. 1B were grown on KS-CDM agarose with 2% glucose, supplemented with either 20 amino acids (20AA), including 2 mM cysteine and 4 mM methionine, or 18 amino acids (18AA), excluding cysteine and methionine; both with and without lipoic acid (LA, an additional source of sulfur). Images were captured after 8–9 days of growth at 34°C with 5% CO_2_ and humidification, with all plates from a single experiment imaged on the same day. Data are from four independent experiments with images from one experiment here and three others in Fig. S5A. + = growth, − = no growth.

In addition to the anabolic modules for biosynthesis, production of cysteine (M00021) and methionine (M00017) (Fig. 4) requires assimilatory sulfate reduction, which takes environmental sulfate and converts it to a usable form in the cell (91). Anvi’o analysis detected stepwise completion scores of only 0.5 for the assimilatory sulfate reduction module (M00176) for *C. glutamicum*^T^, all *C. accolens,* all *C. tuberculostearicum,* most *C. propinquum* (17/19), and 13 of 42 *C*. *pseudodiphtheriticum* genomes. However, *C. glutamicum*^T^ has proven assimilatory sulfate reduction capabilities via the *fpr2-cysIXHDNYZ* locus (92). This operon includes genes for two subunits of a sulfate adenylyltransferase, *cysD* (K00957) and *cysN* (K00956); an APS reductase, *cysH* (K00390); and a sulfite reductase, *cysI* (K00392). Of note, in *E. coli,* two enzymatic steps are required for the release of sulfite from APS: APS kinase (CysC) and PAPS reductase (CysH). Whereas, in *C. glutamicum*, *M. tuberculosis*, and *B. subtilis,* CysH is an APS reductase that directly converts APS to sulfite (92–94). However, the KEGG definition for M00176 currently fails to account for the experimental evidence that a complete assimilatory sulfate reduction module in *C. glutamicum*^T^ does not require an APS kinase. All the strains in our analysis with a stepwise completion score of 0.5 for this module have the same four KOs in the module as *C. glutamicum*^T^ and are, therefore, predicted to perform assimilatory sulfate reduction. However, this module was completely absent in all the *C. pseudodiphtheriticum* strains from the United States and four from Botswana (Fig. 7A), plus 2 *C*. *propinquum* strains, and *C. diphtheriae*^T^. The complete absence of the module for assimilatory sulfate reduction predicts that this subset of *C. pseudodiphtheriticum* strains cannot synthesize methionine or cysteine when sulfate is the only exogenous source of sulfur. To test this, we generated a chemically defined agarose medium supplemented with 18 amino acids (lacking cysteine and methionine) in which 75 mM sodium sulfate was the only exogenous source of sulfur. As predicted *C. pseudodiphtheriticum* MSK311, isolated in Botswana, showed growth under conditions requiring assimilatory sulfate reduction, whereas the U.S. isolate *C. pseudodiphtheriticum* KPL1989 did not (Fig. 7B; Fig. S5A). These findings indicate a recent and geographically localized complete loss of the assimilatory sulfate reduction module M00176 within the *C. pseudodiphtheriticum* phylogeny.

### Nasal *Corynebacterium* species encode for glycine and amino acids involved in nitrogen assimilation

KEGG lacks module definitions for the biosynthesis of six of the standard amino acids: glycine, glutamate, glutamine, aspartate, asparagine, and alanine. Based on gene cluster analysis and the known capabilities of *C. glutamicum^T^*, we predicted that all four nasal *Corynebacterium* species can generate these six amino acids. In *C. glutamicum*^T^, the gene *glyA* encodes the enzyme serine hydroxymethyltransferase (SHMT) that generates glycine from L-serine (95). This essential gene corresponds with K00600, which was present in all the analyzed *Corynebacterium* genomes. Production of the remaining five amino acids is intertwined with nitrogen assimilation, which is well studied in *C. glutamicum*.

Based on genomic comparisons to *C. glutamicum*^T^, we predicted that the four nasal *Corynebacterium* species can assimilate ammonium and synthesize glutamate and glutamine using glutamate dehydrogenase and glutamine synthetase. Tesch et al. propose that glutamine amidotransferase reactions are critical for the flux of NH_4_^+^ into biomass in *C. glutamicum* based on^15^N-ammonium flux measurements showing that *C. glutamicum*^T^ assimilates 72% of the NH_4_^+^ into glutamate using glutamate dehydrogenase and 28% into glutamine via glutamine synthetase (96). Surprisingly, 2-oxoglutarate aminotransferase (GOGAT) does not actively contribute to NH_4_^+^ assimilation in *C. glutamicum*^T^ (96, 97). Moreover, *C. glutamicum*^T^ GOGAT mutations display only a slight increase in doubling time in minimal medium with limiting amounts of ammonium or urea as the nitrogen source, indicating GOGAT is nonessential for ammonium assimilation in *C. glutamicum*^T^ (97). In fact, the genes encoding for the two subunits of the GOGAT in *C. glutamicum*^T^ (*gltBD*) were absent in all of the nasal *Corynebacterium* strains, as were matches to the corresponding KOs (K00265 and K00266). By contrast, using GC analysis, we detected *C. glutamicum*^T^ *gdhA,* encoding glutamate dehydrogenase (K00262), and *glnA,* encoding glutamine synthetase, in all of the nasal strains (except for three *C. propinquum* strains that had a truncated *ghdA*), predicting that the four nasal *Corynebacterium* species can assimilate ammonium and synthesize glutamate and glutamine.

Our analysis also predicted that the four nasal *Corynebacterium* species can all generate aspartate because *C. glutamicum aspT* (which encodes for an aspartate aminotransferase that interchangeably converts glutamate and oxaloacetate to 2-oxoglutarate and aspartate) (87) clustered with genes from all of the nasal *Corynebacterium* strains. The *C. glutamicum aspT* (a.k.a. *aspAT*) was recently recognized as part of the new subgroup 1c of class I aspartate aminotransferases (98), which likely accounts for the lack of KEGG annotation even though the COG and Pfam annotations are consistent with an aspartate aminotransferase domain (COG1167; PF12897.11).

Furthermore, we predicted that the four nasal *Corynebacterium* species do not require exogenous asparagine. We identified a GC including the *C. glutamicum*^T^ *asnB* gene plus genes from all of the analyzed *Corynebacterium* genomes annotated as an asparagine synthase (K01953). However, Hirasawa et al. report that the *C. glutamicum asnB* gene (a.k.a. *ltsA*, GenBank: AB029550.1) fails to complement an *E. coli asnA asnB* double mutant suggesting that it lacks asparagine synthetase activity, which led them to propose that it encodes for a glutamine-dependent amidotransferase that modifies cell wall component(s) involved in lysozyme and temperature sensitivity (99). The putative *asnB* genes of *Mycobacterium tuberculosis* and *Bacillus subtilis* are more similar to *C. glutamicum asnB* than to *E. coli asnB*, suggesting their gene products also lack an asparagine synthase function (99). This agrees with the observation that *M. tuberculosis* relies on the amidation of aspartate-tRNA to incorporate asparagine into proteins (100, 101). Based on these data and that *C. glutamicum^T^* grows in defined medium in the absence of amino acids, we predicted the four nasal *Corynebacterium* species also do not require asparagine.

Finally, we predicted that the four nasal *Corynebacterium* species could synthesize alanine since they all encoded *alaT* (K14260), an aminotransferase that converts pyruvate and glutamate to alanine and 2-oxoglutarate. *C. glutamicum*^T^ encodes two genes, *alaT* and *avtA*, that can both generate alanine via an aminotransferase reaction, and mutants with a deletion of *alaT* have an L-alanine requirement under specific growth conditions (102).

**Fig 8:**
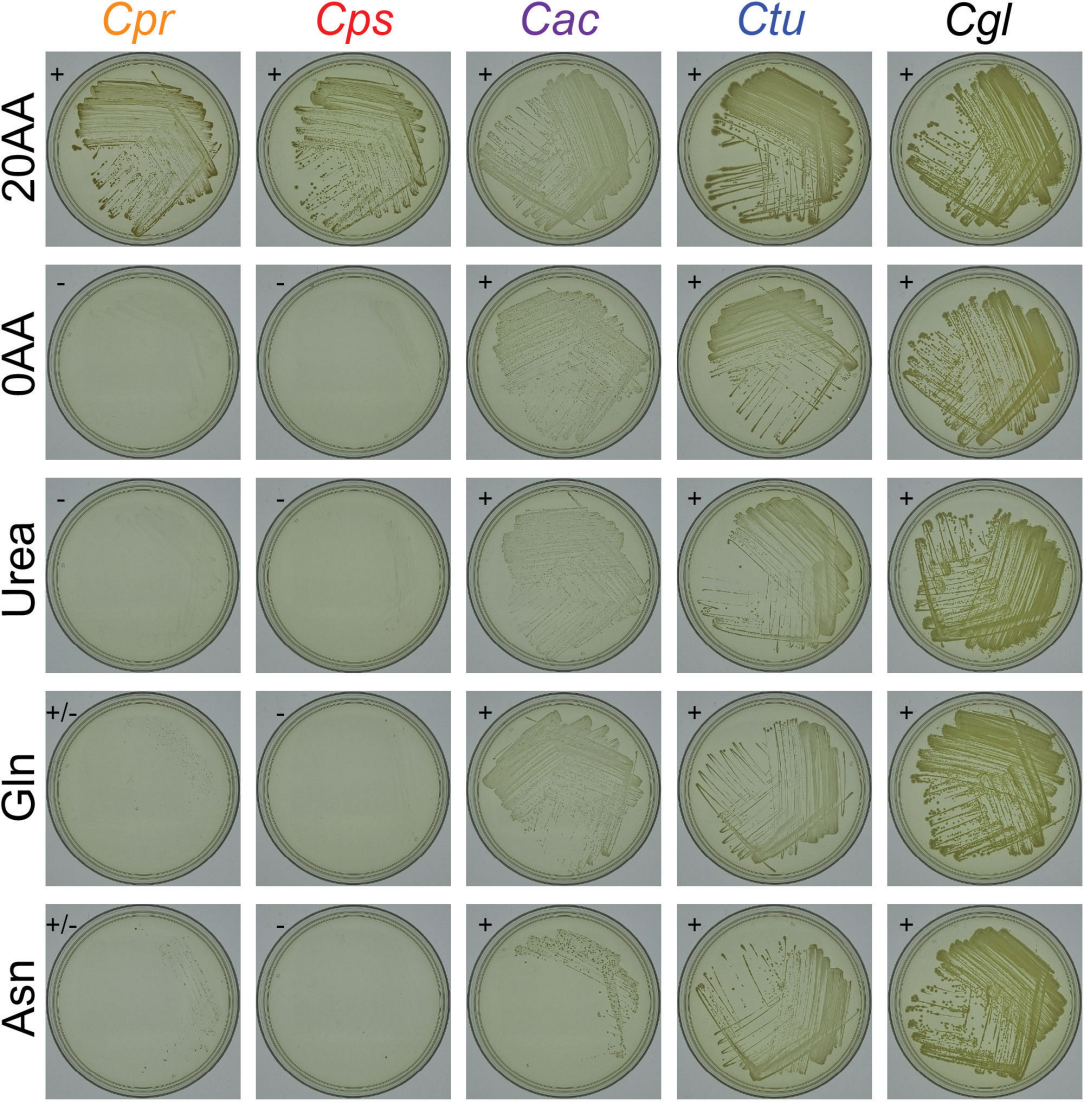
*C. accolens* and *C. tuberculostearicum* grow in the absence of all 20 amino acids on MOPS-buffered CDM agarose. Strains were grown on MOPS-buffered base CDM agarose with 2% glucose supplemented with 20 amino acids (20AA) vs 0 amino acids (0AA), and also in the absence of amino acids supplemented with either 5 mM urea, 10 mM glutamine (Gln), or 10 mM asparagine (Asn) in *n* = 4 independent experiments. Shown are images from one experiment photographed after 8 days of growth at 34°C with 5% CO_2_ and humidification; the remaining data are in Fig. S5B. + = growth, − = no growth. +/− = variation across experiments. Species labels: *C. propinquum* KPL3953 (*Cpr*)*, C. pseudodiphtheriticum* MSK311 (*Cps*)*, C. accolens* KPL1818 (*Cac*)*, C. tuberculostearicum* MSK074 (*Ctu*), and *C. glutamicum* DSM 20300^T^ (*Cgl*).

To test our predictions, we assayed for growth on a base MOPS-buffered CDM agarose supplemented with 2% glucose, with *C. glutamicum*^T^ as a positive control. All four nasal *Corynebacterium* species grew on MOPS-CDM agarose supplemented with all 20 proteinogenic amino acids (Fig. 8; Fig. S5B). Along with *C. glutamicum*^T^, *C. accolens* and *C. tuberculostearicum* both grew in the absence of all 20 amino acids confirming the metabolic predictions, whereas *C. pseudodiphtheriticum* and *C. propinquum* did not. We hypothesized that MOPS-CDM lacking amino acids failed to support the growth in these two species due to nitrogen limitation. Therefore, we tested whether urea, glutamine (File S1), or asparagine, which are all easily bioavailable sources of nitrogen, would restore growth; however, none did so (Fig. 8; Fig. S5B). This leaves open the possibility that both *C. pseudodiphtheriticum* and *C. propinquum* are auxotrophic for at least one amino acid due to either a misannotation or a point mutation(s) resulting in a loss of function, or, alternatively, that under these specific growth conditions, both fail to produce at least one of the required amino acids due to regulatory issues.

### Human nasal *Corynebacterium* species have a broader metabolic capacity for the biosynthesis of amino acids and cofactors/vitamins than *Dolosigranulum pigrum*

**Fig 9:**
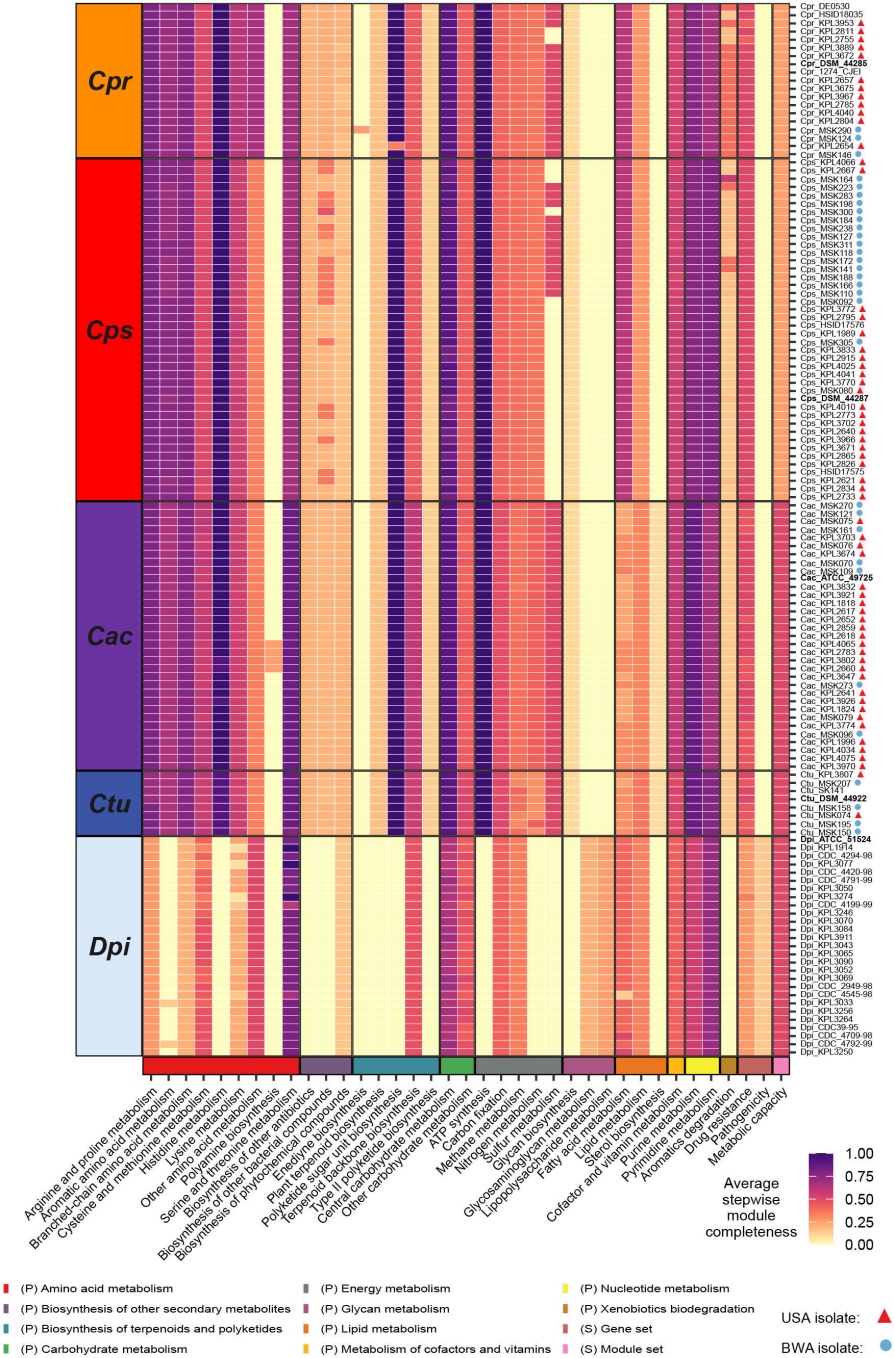
Each of the four nasal *Corynebacterium* species encodes for an increased metabolic capacity compared to *D. pigrum*. This heatmap shows stepwise module completeness averaged by KEGG module subcategory (x-axis labels) for each of four nasal *Corynebacterium* species (average genome size, 2.3–2.5 Mb) and for *D. pigrum* (avg. genome size 1.9 Mb). Average stepwise completion scores were calculated including only modules detected in at least one of the analyzed genomes. The color legend on the bottom represents KEGG module categories and corresponds with the colors in Fig. 4. (P) and (S) indicate Pathway and Signature modules, respectively. Species labels: *C. propinquum* (*Cpr*)*, C. pseudodiphtheriticum (Cps), C. accolens (Cac), C. tuberculostearicum (Ctu*), and *D. pigrum* (*Dpi*).

Many compositional studies of human nasal microbiota show a positive association at the genus level between *Corynebacterium* and *Dolosigranulum* (e.g., references 1, 2, 4, 7, 24, 26, 38, 103). Nasal *Corynebacterium* species can enhance *in vitro* growth yields of *D. pigrum*, a lactic acid-producing bacterium (38). Together with other prior analyses (104), these data indicate *D. pigrum* must access nutrients from its host and its microbial neighbors. As hypothesized, the nasal *Corynebacterium* species with their larger genome sizes (2.3–2.6 Mb) had a greater number of complete KEGG modules per genome than *D. pigrum* (1.9 Mb) (Fig. 9). Using anvi'o, we identified 15 complete modules shared by the majority (≥78%) of the 27 *D. pigrum* strain genomes (Fig. 4, highlighted in light blue). These are for the metabolism of amino acids (2), carbohydrates (8), energy (1), lipids (1), and cofactors and vitamins (3). This is approximately 30% of the number of complete modules found in the majority of each of the four *Corynebacterium* species’ genomes (range 47–56). The module for glycogen biosynthesis (M00854) was the only one found in *D. pigrum* and incomplete in all four *Corynebacterium* species (Fig. 4); however, as described above, *C. tuberculostearicum* produces glycogen (Fig. 6). Of note, each *D. pigrum* genome had at least one CDS annotated as a sialidase (K01186), which can release sialic acid from sialylated glycans found in mucus providing bacteria with carbon and nitrogen (105), whereas there were none in the nasal *Corynebacterium* genomes (Table S3A). Only 10 complete KEGG modules were shared by *D. pigrum* and all 4 *Corynebacterium* species (Fig. 4, highlighted in pale yellow). By contrast, the 4 *Corynebacterium* species shared 12 modules for amino acid metabolism and 4 modules for cofactor/vitamin metabolism in the majority of their genomes that were absent/incomplete in *D. pigrum* (Fig. 4).

## DISCUSSION

Here, we analyzed strain genomes of four common nasal *Corynebacterium* species including those of 87 distinct human nasal isolates collected in Africa and North America across the human lifespan. Phylogenomic analysis showed *C. pseudodiphtheriticum* displays geographically restricted strain circulation. This corresponded with a recent geographically restricted loss of the KEGG module for assimilatory sulfate reduction in strains isolated in the United States since this module was present in the other three species and most *C. pseudodiphtheriticum* strains from Botswana. We confirmed the absence of the *cysIXHDNYZ* operon in the U.S. *C. pseudodiphtheriticum* strains and experimentally demonstrated that a representative U.S. strain failed to grow under conditions requiring assimilatory sulfate reduction, whereas a strain from Botswana grew (Fig. 7). Across the four species, the genomic analysis revealed average genome sizes of 2.3–2.5 Mb, with the average CDS per genome ranging from 2,105 to 2,265 and with 72%–79% of each genome encoding GCs of the shared conservative core genome of the respective species. For each species, the core genome size had leveled off while the pangenome remained open. An informative assignment to a definitive COG category was possible only for approximately 65% of the GCs in the persistent genome and 26%–36% of the GCs in the accessory genome of each species, which points to the need for ongoing experimental research to identify the function of many bacterial GCs. GCs assigned to the COG categories for metabolism were overrepresented in the persistent genome of each species and all four species shared the majority (43 of 58) of complete KEGG modules identified, which implies limited strain- and species-level metabolic variation restricting the possibilities for strains to occupy distinct metabolic niches during the common occurrence of cocolonization of the human nasal passages. We experimentally validated the genome-based metabolic prediction that of the four species only *C. tuberculoste*a*ricum* accumulates intracellular glycogen (Fig. 6), speculating that this might increase the fitness of *C. tuberculostearicum* on skin through rapid glycogen degradation to produce trehalose as an osmoprotectant.

*Corynebacterium* species are often positively associated with *Dolosigranulum* in human nasal microbiota. We found human nasal *Corynebacterium* species have a broader metabolic capacity for biosynthesis of amino acids and cofactors/vitamins than *D. pigrum,* supporting the possibility that *Corynebacterium* might cross-feed or serve as a source of nutrients for *D. pigrum,* and possibly other microbionts, in human nasal microbiota. We experimentally validated that *C. accolens* and *C. tuberculostearicum* both grow on a defined medium lacking amino acids. Our findings combined with data showing that the majority of adults likely host at least two *Corynebacterium* species in their nasal passages point to the importance of future investigation into how *Corynebacterium* species interact with each other and with other microbionts in human nasal microbiota.

By analyzing the extent and distribution of cobamide production in 11,000 bacterial species, Shelton et al. report that 86% of bacteria in their data set have at least 1 of the 15 cobamide-dependent enzyme families, but only 37% are predicted to synthesize their cobamide (74) pointing to widespread interspecies cobamide sharing among bacteria. Swaney et al. find d*e novo* cobamide biosynthesis is enriched in host-associated compared to environment-associated *Corynebacterium* species, with several human skin-associated *Corynebacterium* species encoding complete biosynthesis pathways (61). By contrast, all four nasal *Corynebacterium* species had nearly absent modules for the production of the corrin ring (M00925 or M00924) and the nucleotide loop (M00122) (Table S4C). They also lacked cobamide-dependent enzymes. Furthermore, based on Shelton and colleagues’ analysis of representative genomes, the majority of the most common human nasal bacteria are both likely nonproducers and lack B12-dependent enzymes and B12 pfam binding domains (74), the exception being *C. acnes*, which is a highly prevalent and abundant member of human nasal microbiota, especially in adults (31), and produces the cobamide vitamin B12 (cobalamin) (106).

Limitations of this study include the uneven representation of strains from the United States and Botswana for *C. propinquum* and *C. accolens*; the limited number of *C. tuberculostearicum* strains; the inherent limitations of KEGG annotations; and the predictive nature of genome-based metabolic estimations, some of which still require future experimental validation. To our knowledge, this analysis includes the largest number of strain genomes for *C. pseudodiphtheriticum, C. propinquum*, and *C. accolens* to date, with the aforementioned smaller number for *C. tuberculostearicum*. However, compared to the thousands of strain genomes that have been analyzed for nasal pathobionts, for example, *S. aureus* (107), there are still a limited number of available genomes of nasal *Corynebacterium* species. This highlights the need to build large strain collections of human-associated *Corynebacterium* species to better assess the potential use of these strains for the promotion of human health. Similarly, although we included genomes for strains isolated from two continents and from a range of ages, the geographic sampling was limited compared to the distribution of human populations globally and there has yet to be a systematic large-scale sampling of nasal microbiota across the human lifespan.

Qualitatively, we isolated fewer *C. tuberculostearicum* from nasal swabs than expected based on its prevalence and relative abundance estimated in our earlier reanalysis of 16S rRNA gene V1-V3 sequences from human nasal samples (31). In contrast to using a single gene, here, we assigned isolates to *C. tuberculostearicum* based on WGS with an ANI of ≥95% to the type strain *C. tuberculostearicum* DSM 44922. Only a subset of our isolates from the United States and Botswana with partial 16S rRNA gene Sanger sequences (approximately V1-V3) matching to *C. tuberculostearicum* met this criterion after WGS. This points to the existence of another common nasal *Corynebacterium* species that is closely related to *C. tuberculostearicum*. Recent human skin metagenomic analyses by Salamzade et al. identify metagenome-assembled genomes and the strain genome LK1134 with ANI ≥95% to the genome called “*Corynebacterium kefirresidentii”* (108), which is not a validly published species, and show via phylogenomic analysis these are closely related to *C. tuberculostearicum* (34). Furthermore, using metagenomic analyses, they show sequences matching the “*C. kefirresidentii”* genome are more prevalent on nasal and nearby facial skin sites, whereas *C. tuberculostearicum* is more prevalent and at higher relative abundance on foot-associated skin sites (34). Future work to validly name the species currently identified by the genome called “*C. kefirresidentii*” with designation and deposition of a type strain in publicly accessible stock centers is critical for experiments seeking to identify the function of this species in human nasal microbiota.

Isolation and whole-genome sequencing of multiple strains for microbial species commonly detected in the human microbiome, such as this one, is an ongoing effort across multiple body sites. Collections of genome-sequenced strains from the microbiota are a critical resource for experimentally testing hypotheses generated from metagenomic and metatranscriptomic studies to identify the functions of human microbionts, and mechanisms by which they persist in the microbiome. The Human Oral Microbiome Database (eHOMD; https://www.homd.org/) is an early and ongoing example of a body-site-focused resource for the human microbiome based on a combination of culture-dependent and -independent data (109). Originally focused on the oral cavity, eHOMD now serves the full human respiratory tract (31). More recently, Saheb Kashaf et al. established the Skin Microbial Genome Collection (SMGC), a combined cultivation- and metagenomic-based resource for the human skin microbiome (110). These well-curated, body-site-focused databases serve a critical role in advancing microbiome research, including their importance in shedding light on so-called microbial and metagenomic “dark matter.” The data we presented here serve as a foundational resource for future genomic, metagenomic, phenotypic, metabolic, functional, and mechanistic research on the role of nasal *Corynebacterium* species in human development and health.

## MATERIALS AND METHODS

### Collecting new nasal *Corynebacterium* sp. isolates

The U.S. *Corynebacterium* strains with KPL in their name were collected in Massachusetts, USA, under a protocol approved by the Forsyth Institutional Review Board (FIRB #17-02) as described previously (62). In brief, adults and children participating in scientific outreach events in April 2017 and 2018 performed supervised self-sampling of their nostrils (nasal vestibule) with sterile swabs. They then inoculated their swab onto up to two types of agar medium: (i) brain heart infusion with 1% Tween 80 (BHIT) and 25 microgram/mL fosfomycin (BHITF25) to enrich for *Corynebacterium* sp. and/or (ii) BBL Columbia colistin-nalidixic acid agar with 5% sheep’s blood (CNA BAP). These were incubated at 37°C for 48 h under either atmospheric (BHITF25) or 5% CO_2_-enriched (CNA BAP) conditions. We selected colonies with a morphology typical of nasal *Corynebacterium* species and passed each two to three times for purification on BHIT with 100 ug/mL fosfomycin (BHITF100) at 37°C prior to storage in medium with 15%–20% glycerol at −80°C. (Isolates from 2017 were picked from growth on BHITF100 at 37°C under atmospheric conditions that had been inoculated from sweeps of the original mixed growth on agar medium and stored at −80°C.)

The majority of the *Corynebacterium* strains with MSK in their name were cultured from nasopharyngeal swab samples collected from mothers and infants in a birth cohort study conducted in Botswana, as previously described (13), with a small number also collected from mid-turbinate nasal swab samples from patients cared for within the Duke University Health System (MSK074, MSK075, MSK076, MSK079, and MSK080). Bacteria were cultivated and isolated as previously described (13).

### Selection of nasal *Corynebacterium* isolates for Illumina sequencing

For each KPL-named new isolate, Sanger sequencing (Macrogen, USA) was performed using primer 27F on a V1-V3 16S rRNA gene colony-PCR amplicon (GoTaq Green, Promega) of primers 27F and 519R. We assigned each initial isolate to a genus and a putative species based on the blastn of each sequence against eHOMDv15.1 (31). We then selected a subset of these isolates for whole genome sequencing (WGS). For MSK-named new isolates, all isolates preliminarily assigned to *Corynebacterium* based on MALDI and/or Sanger sequencing of V1-V3 16S rRNA gene underwent WGS.

### Genomic DNA extraction

We extracted genomic DNA (gDNA) from the KPL-named U.S. strains using the MasterPure Gram Positive Genomic DNA Extraction Kit with the following modifications to the manufacturer’s protocol: 10 mg/mL lysozyme treatment at 37°C for 10 minutes and 2 × 30 s bead beat in Lysing Matrix B tubes (MP Biomedicals) at setting 6 on a FastPrep24 (MP Bio) with 1-minute interval on ice. To assess gDNA quality, we performed electrophoresis on 0.5% TBE agarose gel, used a NanoDrop spectrophotometer to quantify 260/280 and 260/230 ratios, and used a Qubit Fluorometric Quantification (Invitrogen) to measure concentration. We extracted gDNA from the MSK-named strains collected in Botswana and North Carolina using the Powersoil Pro extraction kit (Qiagen) following the manufacturer’s instructions. DNA concentrations were determined using Qubit dsDNA high-sensitivity assay kits (Thermo Fisher Scientific).

### Whole-genome sequencing and assembly

For the KPL-named U.S. isolates, Nextera XT (Illumina) libraries were generated from gDNA. Each isolate was sequenced using a paired-end 151-base dual index run on an Illumina Novaseq6000 at the NIH Intramural Sequencing Center. The reads were subsampled to achieve 80× coverage and then assembled with SPAdes (version 3.13.0) (111) and polished using Pilon (version 1.22) (112). For the MSK-named isolates, which are mostly from Botswana, library preparation was performed using DNA Prep Kits (Illumina), and these libraries were sequenced on a NovaSeq 6000 instrument (Illumina) configured for 150 base pair paired-end reads. Adapter removal and read trimming were performed using Trimmomatic version 0.39 (113) to a Phred score of 30 across a 4-bp sliding window. Surviving reads shorter than 70 bp were discarded. The final quality of reads was assessed using FastQC version 0.11.9. Assembly was performed using SPAdes version 3.15.3 (114). The completeness of the genomes was evaluated with checkM version 1.1.3 (115) and all genomes with completeness of less than 95% were discarded. Genomic data are deposited under BioProjects PRJNA842433 for the KPL-named isolates (which are a subset of 94 *Corynebacterium* isolated in MA, USA) and PRJNA804245 for the MSK-named isolates (which are a subset of 71 genomes isolated from Botswana and the Duke University Health System). Table S1A includes NCBI accession IDs.

### Selection of strain genomes for pangenomic analysis

To the 165 assemblies mentioned in the previous section, we added another 16 KPL-named *Corynebacterium* sp. nasal-isolate genomes originally sequenced as part of the HMP and deposited by the Broad at NCBI to consider for analysis (116) (File S1). Furthermore, 31 reference assemblies for relevant *Corynebacterium* species, including the genome of the type strain of *C. propinquum*, *C. pseudodiphtheriticum*, *C. accolens,* and *C. tuberculostearicum* plus three strain genomes of *C. macginleyi,* were downloaded from NCBI using the PanACoTA v1.4.1 (117) “prepare -s” subcommand. We used default parameters such that genomes with MASH distances to the type strain outside of the range 1e-4 to 0.06 were discarded to avoid redundant pairs or mislabeled assemblies and low-quality assemblies based on L90 ≤100, and number of contigs ≤ 999 were filtered out. The collected 212 assemblies were filtered using the “prepare --norefseq” subcommand as above to select higher quality assemblies (L90 ≤100 and number of contigs ≤ 999) and to eliminate redundant genomes defined by a MASH distance <10^−4^ keeping the genome with the highest quality score from each redundant set. Finally, we confirmed the species-level assignment of our nasal isolates, and the nontype reference strains, based on an ANIb (nucleotide) of ≥95% for all shared CDS regions compared to the respective type strain of each species using GET_HOMOLOGUES (see below). For each species, this resulted in a set of distinct strain genomes (including the type strain) that we used for subsequent analyses, which totaled to 104 genomes: 19 *C*. *propinquum* genomes, 43 *C*. *pseudodiphtheriticum* genomes, 34 *C*. *accolens* genomes*,* and 8 *C*. *tuberculostearicum* genomes. Table S1A contains a list of these 104 strain genomes selected for further analysis plus 3 *C*. *macginleyi* reference strains, and Table S1B is the all-by-all MASH distance analysis result of the PanACoTA analysis for all 107 genomes.

### Determination of the conservative core genome

We annotated all bacterial genomes with Prokka version 1.14.6 (118) with default parameters, including gene recognition and translation initiation site identification using Prodigal (119). Then, we used the “./get_homologues.pl” command from GET_HOMOLOGUES version 24082022 (120, 121) to determine a conservative core genome for the selected *Corynebacterium* genomes based on the consensus of three algorithms: bidirectional best-hits (BDBH), cluster of orthologs triangles (COGS) v2.1 (122), and Markov Cluster Algorithm OrthoMCL (OMCL) v2.4 (123) (Fig. S1A; Fig. S2C). Each of the three algorithms reported clustering at the protein level using blastp from NCBI BLAST v2.2 (124) with “-C 90” (min % coverage in BLAST pairwise alignments). The data output created from running the three different clustering algorithms was used to identify the intersection of the core GCs with the command “./compare_clusters.pl” with “-t # of genomes.” We ran this last command twice, with and without the -n flag to generate both nucleotide and protein outputs. Additional methods for genome annotations (https://klemonlab.github.io/CorPGA_Pangenomics/SupplementalMethods_Prokka_Annotations.html) and for the determination of the conservative core genome are available online (https://klemonlab.github.io/CorPGA_Pangenomics/SupplementalMethods_GET_HOMOLOGUES.html).

### Determination of core, soft core, shell, and cloud genomes

For this analysis to identify the core genomes, we dropped the strains Cps_090104 and Cac_ATCC_49726 from their respective species because, although these both passed CheckM analysis, their inclusion in the initial rarefaction analyses caused a splitting of data points toward the lower end resulting in an aberrant lower bound. We separately analyzed the genomes of each *Corynebacterium* species using GET_HOMOLOGUES with the command “./get_homologues.pl” and flag “-t 0” (to get all the possible clusters), first with “-M” (OrthoMCL) and a second time with “-G” (COGS). The COGS and OMCL results were then used with “./compare_clusters.pl” with “-m” (produce intersection pangenome matrices). Then the command “./parse_pangenome_matrix.pl” was used with “-s” (report core, soft core, shell, and cloud clusters), and “-x” (produce the matrix of intersection pangenome clusters) (Fig. S3A). In-depth code and descriptions to determine the core, soft core, shell, and cloud genomes are available online (https://klemonlab.github.io/CorPGA_Pangenomics/SupplementalMethods_GET_HOMOLOGUES.html).

### Calculating the conservative core genome for each species with the addition of an outgroup

We chose the type strain genome of the most closely related species from the phylogenomic tree in Fig. S1B to serve as the outgroup in each species-specific phylogeny with *C. pseudodiphtheriticum* DSM 44287 for the *C. propinquum* phylogeny, *C. propinquum* DSM 44285 for the *C. pseudodiphtheriticum* phylogeny*, C. macginleyi* CCUG 32361 for the C. *accolens* phylogeny, and *C. accolens* ATCC 49725 for the *C. tuberculostearicum* phylogeny. The addition of an outgroup resulted in four new data sets with *n* + 1 total genomes, where n denotes the number of strain genomes for each species. Each species-plus-outgroup data set of genomes was analyzed as before using the commands “. /get_homologues.pl” (with BDBH, COG triangles, and OrthoMCL) and “./compare_clusters.pl,” but now with the outgroup genome included (Fig. S2D). For the construction of single-species trees (Fig. 1), we then combined this smaller conservative core of GCs shared between the species and its outgroup (shared intersection of each Venn diagram in Fig. S2D) with the subset of GCs belonging only to the conservative core of each species (which is the intersection of each Venn diagram in Fig. S2C minus the intersection of those in Fig. S2D). The code used to do this is available online (https://klemonlab.github.io/CorPGA_Pangenomics/SupplementalMethods_GET_HOMOLOGUES.html).

### Construction of phylogenomic trees

We used GET_PHYLOMARKERS v.2.2.9.1 to concatenate and align the single-copy core GCs for each phylogeny (125). The command “run_get_phylomarkers_pipeline.sh” was run with the flags: “-R 1 -t DNA -k 0.7 -m 0.7” on both the protein and nucleotide GET_HOMOLOGUES outputs. The flag “-R” was used to select optimal markers for phylogenomics; “-t” for whether the input is DNA or protein; “-k” for kde stringency; and “-m” for the minimum average support values for trees to be selected. The codon fasta alignments generated by GET_PHYLOMARKERS were analyzed with IQ-TREE v2.1.3 (126) with: “-p,” [uses edge linked partition model and ModelFinder functions (127–129)], “-alrt 1000” (perform replicate SH-like approximate likelihood ratio test) and “-B 1000” (number of ultrafast bootstrap replicates). The phylogenetic tool iTOL v6 (130) was used to visualize, scale, edit, annotate names, and root the tree at the midpoint for each phylogeny. Detailed code and methods to create these phylogenies are available online (https://klemonlab.github.io/CorPGA_Pangenomics/SupplementalMethods_GET_HOMOLOGUES.html).

### Rarefaction analysis and ANI across strain genomes

For each species, with GET_HOMOLOGUES, we used rarefaction analysis to estimate whether the core genome and pangenome were closed or open (Fig. 2). We modified “$MIN_PERSEQID_HOM” and “$MIN_COVERAGE_HOM” values to equal 50 (inside “marfil_homology.pm”) so only protein sequences with identity and coverage ≥50% will be called homologs. Again, we decided to eliminate the two genomes Cac_ATCC_49726 and Cps_090104 from their respective species, because when preliminarily included the graph for each species’ core genome size became bifurcated while conducting random sampling. This is because the core genome size of each of the excluded strains was considerably lower, resulting in a misleading lower bound. The clustering was redone for each species using “./get_homologues.pl” and the following parameters: “-C 90,” “-c,” (genome composition analysis), and “-M” for OMCL. The rarefaction curve .tab files were produced from the “-c” flag. Rarefaction .tab files for each species were plotted into svg files using “/plot_pancore_matrix.pl” with flags “-f core_both” (displays both Tettelin and Willenbrock curves for core genome), and “-f pan” (curve for estimating pangenome size).

Blastn instead of blastp was used to report GCs for ANI heat plots. To generate the core and all the shared CDS regions ANI .tab files, we used the command “./get_homologues.pl” with flags: “-d,” “-A,” “-t,” “-a,” “-M.” Furthermore, to plot the ANI heatmaps, the “./plot_matrix_heatmap.sh” command was used.

### Functional analysis of four *Corynebacterium* pangenomes using anvi’o and PPanGGOLiN

The pangenome for each *Corynebacterium* species was analyzed using anvi’o v8 (64, 131). We used a workflow that allowed us to import Prokka-annotated genomes into anvi’o (https://klemonlab.github.io/CorPGA_Pangenomics/SupplementalMethods_Prokka_Annotations.html), followed by the addition of functional COG annotations using the “anvi-run-ncbi-cogs” command with --sensitive flag (runs sensitive version of DIAMOND [132]) and the 2020 updated COG20 database (133, 134). Various annotations were also applied to each genome db file, such as KEGG/KOfam (135, 136), Pfam (137, 138), and hmm-hits (139). The pangenome for each species was computed with the “anvi-pan-genome” program (flags: --mcl-inflation 10, and --use-ncbi-blast) using blastp search (140), muscle alignment (141), “minbit heuristic” (142) to filter weak hits, and the MCL algorithm (143). A combined pangenome was also computed for 107 strains: the 102 nasal *Corynebacterium* genomes selected for metabolic analysis (Cac_ATCC_49726 & Cps_090104 were excluded) plus 5 *Corynebacterium* listed in Table S1D. This high-level pangenomic analysis was used for gene cluster-level comparisons of the KEGG results. The functional and the geometric homogeneity index, and the rest of the layers shown within the anvi’o pangenome *Corynebacterium* figures were determined from the standard anvi’o pangenomic pipeline (https://merenlab.org/2016/11/08/pangenomics-v2/). The core (genes contained in all genomes), soft core (genes contained in 95% of the genomes), shell (genes contained in several genomes), and cloud (genes present in only a few genomes) assignments from GET_HOMOLOGUES were uploaded into the anvi’o pangenome for each species by creating bins in the anvi’o interactive interface. We also manually rearranged the order of strains in each species-specific analysis to match the order in our species-specific phylogenomic trees and imported our phylogenies into the anvi’o figures. This facilitated visualizing the strain genomes from an evolutionary standpoint and identifying GCs that might be unique to a strain or a group of strains in a clade. The code used to generate the anvi’o pangenomic analysis can be found at https://klemonlab.github.io/CorPGA_Pangenomics/SupplementalMethods_Anvio.html. We then exported the summary files from the anvi’o pangenomic analyses to synchronize gene cluster identities with PPanGGOLiN v1.2.74 (67) (Table S2) (for detailed code, see https://klemonlab.github.io/CorPGA_Pangenomics/SupplementalMethods_PPanGGOLiN.html). GCs were defined as persistent or accessory by PPanGGOLiN and then we used an in-house R script (https://github.com/KLemonLab/CorPGA_Pangenomics/blob/main/SupplementalMethods_COGS.Rmd) to clean up and retrieve informative COG20 annotated GCs and to generate the functional enrichment plots shown in Fig. 3 and Fig. S4.

### Estimation of metabolic capabilities using anvi’o v8

We then used “anvi-estimate-metabolism” (144) to estimate gene enzymatic functions (Table S3A) and the completeness of KEGG modules in each of the four *Corynebacterium* species (using the selected 102 strains), in each of 27 *D. pigrum* strain genomes (62), and in each reference genome of the nine other species listed in Table S1D, using their original NCBI annotations. The complete KEGG modules estimation output in tabular format is provided in Table S4A, including stepwise and pathwise completeness scores, and, for easier readability, in Table S4B as a matrix summarizing module stepwise completeness by genome. Table S4C includes the module estimations averaged by species. For the data summaries presented in Fig. 4 and 5, we excluded modules that were complete in <12.5% of the strains of each species, across all analyzed species. Additional detailed methods and code, including the code for generating Fig. 4 and 9, are available online (https://klemonlab.github.io/CorPGA_Pangenomics/SupplementalMethods_Anvio.html).

### Determining KEGG module enrichment

We used “anvi-display-functions” and “anvi-compute-metabolic-enrichment” (145), with a module stepwise completeness score of 1 for the latter, to identify individual KOs (Table S3B) and KEGG modules (Table S4D) enriched across the four *Corynebacterium* species. Modules with an adjusted *q*-value <1e−9 were considered enriched in their associated group. Modules both with an adjusted *q*-value >1e−9 and complete in at least 87% of the analyzed genomes were considered shared. Additional detailed methods and code are available online (https://klemonlab.github.io/CorPGA_Pangenomics/SupplementalMethods_Anvio.html).

### Bacterial strains and growth conditions

*C. propinquum* KPL3953, *C. pseudodiphtheriticum* MSK311 and KPL1989, *C. accolens* KPL1818, *C. tuberculostearicum* MSK074, and *C. glutamicum* DSM 20300^T^ were each grown from a −80°C freezer stock on Difco Brain Heart Infusion (BHI) agar medium supplemented with 1% Tween 80 (BHIT) for 36–48 hours at 34°C in a 5% CO_2_ CellXpert C170 incubator (Eppendorf, # 6734010015) with a humidification pan. Subsequently, for each strain, we carefully collected 10–20 single colonies from BHIT agar and pooled these for resuspension in individual 5 mL Eppendorf tubes containing 4.5 mL of either phosphate buffered saline (PBS) for the glycogen experiments or Earle’s Balanced Salt Solution (EBSS) for the growth assays with amino acid supplementation.

### MOPS-buffered chemically defined medium base

We used a base chemically defined medium (CDM) buffered with 3-(N-morpholino)propanesulfonic acid (MOPS) into which we supplemented specific concentrations of glucose, varying amino acids, and/or other supplements, as detailed below, to generate sterile chemically defined media. MOPS-buffered base CDM is a 2× solution of a Teknova medium prepared from Teknova 10× MOPS buffer (M2101), Teknova 10× AGCU solution (M2103), Teknova 100× Vitamin Stock I (V1015), and Teknova 0.132 M potassium phosphate dibasic (M2102) plus 1,000× ferric chloride, 400× lipoic acid, and Tween 80 with the following final concentrations: 80 mM MOPS, 8 mM tricine, 2.64 mM potassium phosphate dibasic anhydrous, 3 mM potassium hydroxide, 0.398 mM adenine, 0.398 mM cytosine, 0.398 mM uracil, 0.398 mM guanine, 0.02 mM ferrous (iron II) sulfate heptahydrate, 19 mM ammonium chloride, 0.552 mM potassium sulfate, 0.001 mM calcium chloride dihydrate, 1.05 mM magnesium chloride hexahydrate, 100 mM sodium chloride, 5.84E−07 mM ammonium molybdate, 8.00E−05 mM boric acid, 6.04E−06 mM cobalt chloride hexahydrate, 1.92E−06 mM copper sulfate pentahydrate, 1.62E−05 mM manganese (II) chloride tetrahydrate, 1.95E−06 mM zinc sulfate heptahydrate, 0.2 mM ferric chloride hexahydrate, 0.408 mM choline chloride, 0.406 mM nicotinic acid, 1.46E−02 mM pyridoxine HCl, 3.94E−02 mM calcium pantothenate, 0.3732 mM PABA, 0.1048 mM thiamine HCl, 8.18E−04 mM biotin, 1.48E−05 mM cyanocobalamin, 3.16E−04 mM folic acid, 2.06E−02 mM riboflavin, 0.0192 mM lipoic acid, 0.1% Tween 80. The pH of the medium is 7 to 7.5.

### Twenty amino acid mix

We prepared a 5× solution mixture of 20 amino acids in MilliQ H_2_O such that the final concentration of each after the addition to base CDM was as follows: 1.6 mM L-alanine, 10.4 mM L-arginine, 0.8 mM L-asparagine, 0.8 mM L-aspartic acid, 0.2 mM L-cysteine, 1.2 mM L-glutamic acid, 1.2 mM L-glutamine, 1.6 mM L-glycine, 0.4 mM L-histidine, 0.8 mM L-isoleucine, 1.6 mM L-leucine, 0.8 mM L-lysine, 0.4 mM L-methionine, 0.8 mM L-phenylalanine, 0.8 mM L-proline, 20 mM L-serine, 0.8 mM L-threonine, 0.2 mM L-tryptophan, 0.4 mM L-tyrosine, 1.2 mM L-valine.

### Determination of intracellular glycogen concentration

Bacteria were resuspended in PBS as described above then washed twice by centrifugation at 16,000 RCF (relative centrifugal force) at 4°C for 10 minutes using a 5430R centrifuge (Eppendorf # 022620689) and resuspended in 4.5 mL of PBS. Next, we used PBS to adjust the OD_600_ to 2 for each strain and then inoculated 1.25 mL of each as a monoculture into 50 mL base phosphate-buffered CDM supplemented with 5% glucose and the 20 amino acid mix in a 250 mL flask. Cultures were grown in a shaking incubator at 250 RPM and 34°C. At 24, 28, 32, and 48 hours, we measured the OD_600_ and collected 4 mL aliquots of each strain. Samples were centrifuged, resuspended in PBS, and washed twice as above. Of note, no clumping was observed either during growth in shaken medium or during the PBS washes. After washing, the samples were resuspended in 400 µL PBS, and the OD_600_ was measured and adjusted with PBS to ~0.5 to assay an equal biomass per strain. We recorded the OD_600_ values and used these to normalize the assayed glycogen per unit of OD_600_. A 300 µL aliquot of each strain was then combined with 150 µL of HCl in a 2 mL lysis matrix B tube (MP Biomedicals, # 116911050-CF) and heated at 95°C for 5 minutes. To lyse the bacteria, we bead beat the samples 3 times at 6.5 m/s for 45 seconds each time using a FastPrep-24 5G (MP Biomedicals # 116005500). After mechanical lysis, 150 µL of Tris buffer was added to each tube. The samples were then centrifuged at 16,000 RCF at 4°C for 20 minutes. The supernatant was recovered and 25 µL of each sample was aliquoted into six separate wells per strain in a white 96-well plate with clear bottom (Greiner Bio-One # 655074) and assayed for glycogen with the Glycogen-Glo Assay kit (Promega # J5051). Per the manufacturer’s instructions, we prepared a glycogen titration curve with twofold serial dilutions in PBS from 20 to 0 µg/mL and we added 25 µL of each of these standards to the 96-well plate for each time point: one set of standards for the glucoamylase assay and one as the control. We then added either 25 µL of glucoamylase solution or buffer alone to each sample (three technical replicates each) and standard well. Plates were incubated at room temp for 1 hour to allow the glucoamylase to digest glycogen into free glucose. Following incubation, 50 µL of glucose detection reagent was added to each well. Luminescence was measured after 70 minutes using a BioTek Synergy H1 microplate reader (Agilent # SH1M-SN) with default settings. The average luminescence for glucoamylase-treated and buffer-only samples was calculated from three technical replicates for each strain at each timepoint for each independent experiment. The average value from the buffer-only samples was subtracted from the glucoamylase-treated samples for each sample to correct for background levels of free glucose. The glycogen titration curve was derived by subtracting the luminescence of the buffer-only negative controls from the glucoamylase-positive standards to establish a best-fit regression curve. The average differences in luminescence values from bacterial samples with and without glucoamylase treatment were input into the corresponding best-fit curve equation to determine the total glycogen concentration (µg/mL) per sample. These were normalized to µg/mL per OD_600_ of the bacterial suspension measured before mechanical lysis to approximate the glycogen concentration per cell biomass of each strain. Statistics were performed using a linear mixed model with species as the fixed effect and time point as a random effect fit by restricted maximum likelihood with *t*-tests using Satterthwaite’s method with R packages lme4 (146) and afex. Additional detailed methods and code, including the code for generating Fig. 6A and B, are available online https://klemonlab.github.io/CorPGA_Pangenomics/SupplementalMethods_Glycogen.html.

### Sequence alignment for the *fpr2-cysIXHDNYZ* operon

We manually inspected KO assignments for all the enzymes encoded in the *C. glutamicum fpr2-cysIXHDNYZ* operon (92) that were absent from the KEGG definition of the assimilatory sulfate reduction module (M00176). Genes annotated as K00528 (*fprA*) were identified in all the *Corynebacterium* genomes. A manual exploration of the gene annotations in the genomic region around the putative *fprA* gene in all *C. pseudodiphtheriticum* strains identified *hchA* as a common gene downstream of the *fpr2-cysIXHDNYZ* operon. We used BEDTools (147, 148) to extract the genomic sequence flanked by *fprA* and *hchA* and performed multiple sequence alignment using MAFFT (149, 150). AliView (151) was used for sequence inspection and generation of a reverse-complemented alignment. The final visualization in Fig. 7A was generated in R with the package msavisr. Additional detailed methods and code are available online https://klemonlab.github.io/CorPGA_Pangenomics/SupplementalMethods_Anvio.html

### Assay for growth under conditions requiring assimilatory sulfate reduction

We generated a phosphate-buffered version of base CDM with 75 mM (f.c.) sodium sulfate as the only source of sulfur (KS-CDM) by replacing the Teknova MOPS buffer with a phosphate buffer consisting of potassium phosphate monobasic, potassium phosphate dibasic, and sodium phosphate dibasic and trace minerals (see below) since *C. glutamicum* can reportedly use MOPS as a source of sulfur (152). We supplemented the KS-CDM base with 2% glucose (Teknova 20% Glucose solution G0520) and an 18 amino acid mix, excluding cysteine and methionine, to generate KS-CDM 0.76% agarose. We also assayed for growth on KS-CDM agarose supplemented with a 75× cysteine and methionine solution to final concentrations of 2 mM cysteine and 4 mM methionine. Both conditions were tested with and without lipoic acid, as a potential additional sulfur source. Prior to inoculation for each experiment, each bacterial strain resuspended in EBSS (as described above) was centrifuged at 10,000 RCF for 10 minutes and then subjected to three washes of 4.5 mL EBSS. After the final wash, cells were resuspended in 500 µL EBSS to achieve a visibly turbid solution and 15 µL from the resuspension of each strain was inoculated and struck for single colonies onto KS-CDM 0.76% agarose (SeaKem LE Agarose, # 50004) prepared with the different variations and incubated at 34°C in a 5% CO2 incubator with a humidification pan. We used agarose because of its higher purity compared to agar. The KS-CDM agarose plates were imaged at 8–9 days to assess bacterial growth with each supplementation, with all plates from a single experiment imaged on the same day. We performed four independent experiments (Fig. 7B; Fig. S5A). KS-CDM is a 2× solution of a Teknova medium prepared from Teknova 10× AGCU solution (M2103), and Teknova 100× Vitamin Stock I (V1015), plus 50,000× Trace Minerals I and 50× Trace Minerals II (File S1), 1,000× ferric chloride, 1,000× ferrous chloride, 10× phosphates, 10× NaCl, 100× tricine, 25× sodium sulfate, 400× lipoic acid, and Tween 80. Final concentrations in KS-CDM were as follows: 37 mM potassium phosphate monobasic, 109 mM potassium phosphate dibasic, 63 mM sodium phosphate dibasic, 0.398 mM adenine, 0.398 mM cytosine, 0.398 mM uracil, 0.398 mM guanine, 8 mM tricine, 75 mM sodium sulfate, 6.04E-06 mM cobalt chloride hexahydrate, 1.92E-06 mM copper chloride dihydrate, 1.62E-05 mM manganese chloride tetrahydrate, 5.84E-07 mM ammonium molybate, 1.95E-06 mM zinc chloride, 0.00008 mM boric acid, 19 mM ammonium chloride, 0.001 mM calcium chloride, 1.05 mM magnesium chloride, 0.2 mM ferric (Iron III) chloride hexahydrate, 0.02 mM ferrous (Iron II) chloride tetrahydrate, 35 mM sodium chloride, 1.6 mM L-alanine, 10.4 mM L-arginine, 0.8 mM L-asparagine, 0.8 mM L-aspartic Acid, 1.2 mM L-glutamic acid, 1.2 mM L-glutamine, 1.6 mM L-glycine, 0.4 mM L-histidine, 0.8 mM L-isoleucine, 1.6 mM L-leucine, 0.8 mM L-lysine, 0.8 mM L-phenylalanine, 0.8 mM L-proline, 20mM L-serine, 0.8 mM L-threonine, 0.2 mM L-tryptophan, 0.4 mM L-tyrosine, 1.2 mM L-valine, and 0.1% Tween 80.

### Amino acid auxotrophy assays

To test for amino acid auxotrophy, we used MOPS-buffered base CDM 0.76% agarose supplemented with 2% glucose and either lacking amino acids (0AA) or supplemented with one of the following: the 20 amino acid mix, 5 mM urea, 10 mM glutamine, or 10 mM asparagine. Bacterial inocula were prepared and growth assays were performed as described above in testing for assimilatory sulfate-reducing ability. The MOPS-CDM agarose plates were imaged at 8 days to assess bacterial growth with each supplementation. We performed four independent experiments (Fig. 8; Fig. S5B).

## Data Availability

All genomes are available from NCBI in Bioprojects PRJNA842433 and PRJNA804245. Table S1 lists genome accession numbers. Code used is in our GitHub repository https://github.com/KLemonLab/CorPGA_Pangenomics.

## SUPPLEMENTAL MATERIAL

File S1 msystems.01132-24-s0001.pdf Supplemental text.

Figure S1 msystems.01132-24-s0002.pdf Phylogenomic trees.

Figure S2 msystems.01132-24-s0003.pdf Pairwise ANIs and conservative core GCs.

Figure S3 msystems.01132-24-s0004.pdf Pangenome estimates.

Figure S4 msystems.01132-24-s0005.pdf COG analysis of accessory genome by strain.

Figure S5 msystems.01132-24-s0006.pdf Additional data related to Fig. 7 and 8.

Table S1 msystems.01132-24-s0007.xlsx Bacterial strain genome information.

Table S2 msystems.01132-24-s0008.xlsx PPanGGOLiN assignment of GCs to the persistent or accessory genome.

Table S3 msystems.01132-24-s0009.xlsx KO metabolic estimations and enrichment analysis by anvi'o.

Table S4 msystems.01132-24-s0010.xlsx KEGG module estimations and enrichment analysis by anvi'o.

ASM does not own the copyrights to Supplemental Material that may be linked to, or accessed through, an article. The authors have granted ASM a non-exclusive, world-wide license to publish the Supplemental Material files. Please contact the corresponding author directly for reuse.
