# Supplementary material for: Metabolic capabilities are highly conserved among human nasal-associated Corynebacterium species in pangenomic analyses
Source: mSystems. 2024 Nov 7;9(12):e01132-24. doi: 10.1128/msystems.01132-24 (PMC11651106; doi:10.1128/msystems.01132-24)
Supplement: File S1 — Supplemental text. [file msystems.01132-24-s0001.pdf]

SUPPLEMENTAL FILE S1

Species assignment for *Corynebacterium* sp. KPL# previously submitted to NCBI. The Broad Institute originally sequenced a set of *Corynebacterium* strains we had isolated from human nostril swabs, and we included in our current analyses any of these that were assigned to one of the four species of interest (1). The species assignment for each of these based on phylogenomic analyses (**Figs. 1** and **S1**) and ANI (**Figs. S2A-B**) are as follows: *C. accolens* KPL1818; *C. accolens* KPL1824; *C. accolens* KPL1996 (*C. accolens* KPL1986, KPL1998, and KPL2004); and *C. pseudodiphtheriticum* KPL1989 (*C. pseudodiphtheriticum* KPL1995). Isolates in parentheses are genomes listed in NCBI that have a MASH distance ≤ 10^{-4} to the strain genome listed immediately before the parentheses.

Comparing core genomes and pangenomes predicted by GET_HOMOLOGUES and anvi'o. The difference in the outputs of gene clusters (GCs) from GET_HOMOLOGUES (consensus of OMCL and COGS algorithms) and anvi'o were measured using the percent difference formula $\frac{|V1-V2|}{\left[\frac{(V1+V2)}{2}\right]} \times 100 = \%\, difference$. Core and pangenomes sizes for both the pangenomic platforms are listed below for comparison, with their percentage differences.

**Species**	**Core genome**			**Pangenome**		
	GH	anvi'o	% diff.	GH	anvi'o	% diff.
C. propinquum	1624	1824	11.6%	3777	3108	19.4%
C. pseudodiphtheriticum	1515	1714	12.3%	4590	3590	24.4%
C. accolens	1688	1904	12.0%	4220	3427	20.7%
C. tuberculostearicum	1810	1915	5.6%	3232	2907	10.6%

L-Glutamine is also an excellent nitrogen source in *C. glutamicum*. Although the GOGAT enzyme is the main pathway involved in glutamine assimilation, it is not required for this, since in a *C. glutamicum* GOGAT deletion mutant, a glutaminase in conjunction with glutamate dehydrogenase appears to contribute to glutamine utilization, although primarily as a carbon and energy source (2, 3). In all the analyzed *Corynebacterium* genomes, except for *C. diphtheriae*^{T}, we identified genes annotated as a glutaminase (K01425) corresponding to the *C. glutamicum*^{T} *glsK* gene, which hydrolyzes glutamine producing ammonium and glutamate. Glutamine is also essential to produce carbamoyl phosphate, a precursor for the arginine and pyrimidine synthesis pathways. In *C. glutamicum* the glutamine-dependent carbamoyl phosphate synthetase II (CPS_II) encoded by the *carAB* operon converts HCO_3^-, ATP, and glutamine to carbamoyl phosphate (4). We identified K01956 and K01955 (*carA* and *carB*) annotations in all analyzed *Corynebacterium* species. Based on these findings, we predicted that addition of glutamine might improve the growth of nasal *Corynebacterium* species in defined medium lacking amino acids via enzymatic activities that use glutamine as a precursor, including, but probably not limited to, glutaminase, carbamoyl phosphate, and/or glutamine amidotransferase enzymes.

Trace Minerals Solutions. The Trace Minerals I 50000x stock consists of the following: 0.302 mM cobalt chloride hexahydrate, 0.096 mM copper chloride dihydrate, 0.81 mM manganese chloride tetrahydrate, 0.0292 mM ammonium molybdate, 0.0975 mM zinc chloride, 4 mM boric acid. The Trace Minerals II 50x stock consists of the following: 950 mM ammonium chloride, 0.05 mM calcium chloride, 52.5 mM magnesium chloride.

REFERENCES

1. Human Microbiome Jumpstart Reference Strains C, Nelson KE, Weinstock GM, Highlander SK, Worley KC, Creasy HH, Wortman JR, Rusch DB, Mitreva M, Sodergren E, Chinwalla AT, Feldgarden M, Gevers D, Haas BJ, Madupu R, Ward DV, Birren BW, Gibbs RA, Methe B, Petrosino JF, Strausberg RL, Sutton GG, White OR, Wilson RK, Durkin S, Giglio MG, Gujja S, Howarth C, Kodira CD, Kyrpides N, Mehta T, Muzny DM, Pearson M, Pepin K, Pati A, Qin X, Yandava C, Zeng Q, Zhang L, Berlin AM, Chen L, Hepburn TA, Johnson J, McCorrison J, Miller J, Minx P, Nusbaum C, Russ C, Sykes SM, Tomlinson CM, et al. 2010. A catalog of reference genomes from the human microbiome. Science 328:994-9.
2. Rehm N, Georgi T, Hiery E, Degner U, Schmiedl A, Burkovski A, Bott M. 2010. L-Glutamine as a nitrogen source for Corynebacterium glutamicum: derepression of the AmtR regulon and implications for nitrogen sensing. Microbiology (Reading) 156:3180-3193.
3. Buerger J, Rehm N, Grebenstein L, Burkovski A. 2016. Glutamine metabolism of Corynebacterium glutamicum: role of the glutaminase GlsK. FEMS Microbiol Lett 363.
4. Wang Q, Jiang A, Tang J, Gao H, Zhang X, Yang T, Xu Z, Xu M, Rao Z. 2021. Enhanced production of L-arginine by improving carbamoyl phosphate supply in metabolically engineered Corynebacterium crenatum. Appl Microbiol Biotechnol 105:3265-3276.
