# Supplementary material for: Metabolic capabilities are highly conserved among human nasal-associated Corynebacterium species in pangenomic analyses
Source: mSystems. 2024 Nov 7;9(12):e01132-24. doi: 10.1128/msystems.01132-24 (PMC11651106; doi:10.1128/msystems.01132-24)
Supplement: Figure S4 — COG analysis of accessory genome by strain. [file msystems.01132-24-s0005.pdf]

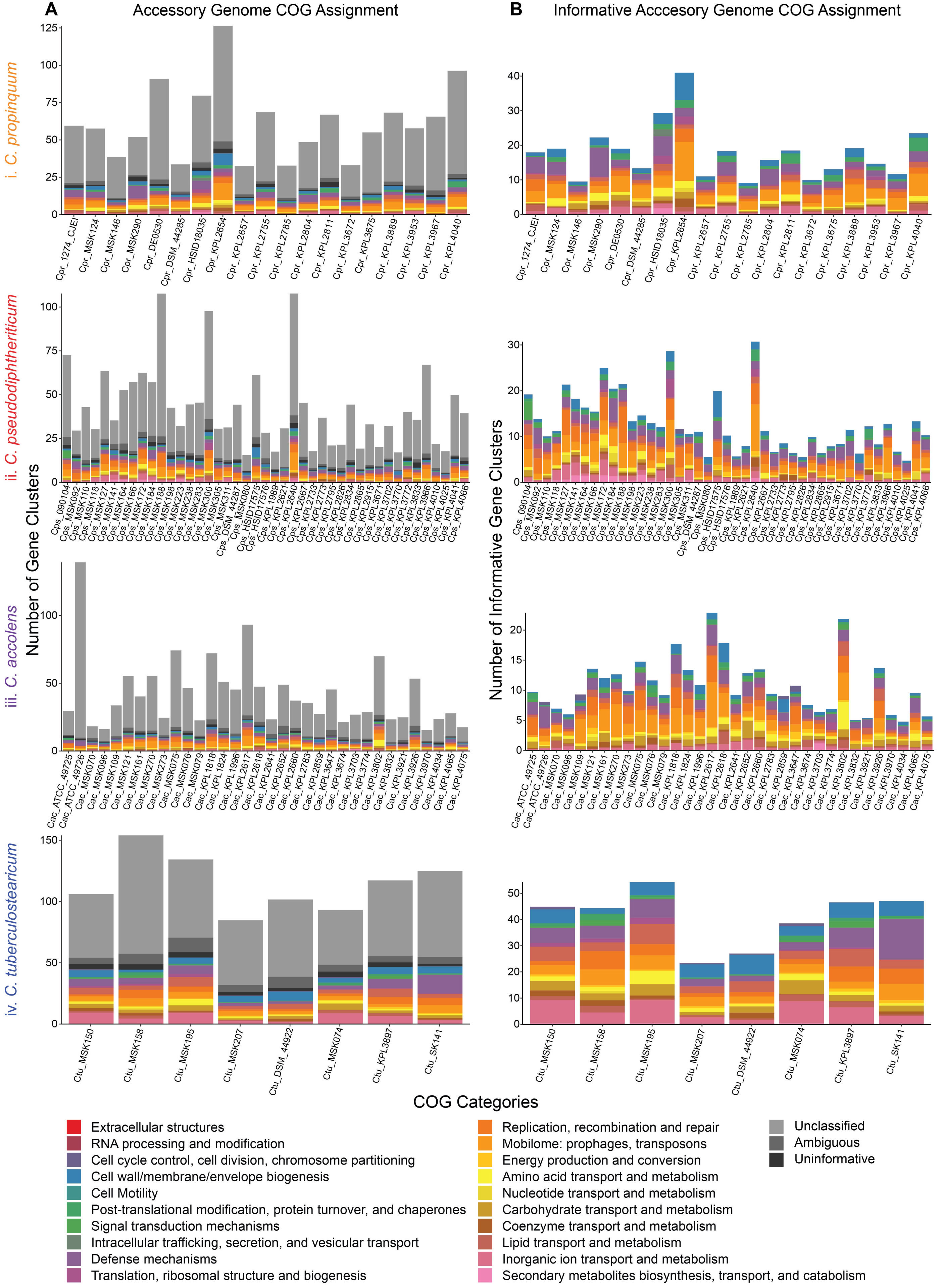

A
Accessory Genome COG Assignment
B
Informative Acccesory Genome COG Assignment
i. C. propinquum
ii. C. pseudodiphtheriticum
iii. C. accolens
iv. C. tuberculostearicum
Number of Gene Clusters
Number of Informative Gene Clusters
COG Categories
Extracellular structures
RNA processing and modification
Cell cycle control, cell division, chromosome partitioning
Cell wall/membrane/envelope biogenesis
Cell Motility
Post-translational modification, protein turnover, and chaperones
Signal transduction mechanisms
Intracellular trafficking, secretion, and vesicular transport
Defense mechanisms
Translation, ribosomal structure and biogenesis
Replication, recombination and repair
Mobilome: prophages, transposons
Energy production and conversion
Amino acid transport and metabolism
Nucleotide transport and metabolism
Carbohydrate transport and metabolism
Coenzyme transport and metabolism
Lipid transport and metabolism
Inorganic ion transport and metabolism
Secondary metabolites biosynthesis, transport, and catabolism
Unclassified
Ambiguous
Uninformative

Figure S4. The size of the accessory genome varies between strains within each nasal *Corynebacterium* species. We used anvi'o to identify COG functional annotations and PPanGGOLiN to assign GCs to the persistent vs. accessory genome. (**A**) For each species (**i-vi**), most of the GCs in each strain's accessory genome had either no annotation ("unclassified"), an ambiguous COG categorization ("ambiguous"), or belonged to an uninformative S or R COG category ("uninformative"). The calculated average percentages for unclassified, ambiguous, and uninformative categories per species were (**i**) 65.2%, 3.5%, and 2.4% for *C. propinquum*; (**ii**) 62.8%, 4.4%, and 2.9% for *C. pseudodiphtheriticum*; (**iii**) 67.6%, 4.7%, and 2.0% for *C. accolens*; and (**iv**) 55.1%, 6.2%, and 3.1% for *C. tuberculostearicum.* The accessory genome of *C. accolens* had the highest percentage of COGs belonging to these three categories and *C. tuberculostearicum* had the lowest. Averages of the informative assigned COG annotations (colored categories) in the accessory genome were calculated for each species as follows: (**i**) 28.9% for *C. propinquum*, (**ii**) 29.9% for *C. pseudodiphtheriticum*, (**iii**) 25.7% for *C. accolens*, and (**iv**) 35.6% for *C. tuberculostearicum*. (**B**) There was a relatively proportional distribution of accessory informative functional GCs among each set of genomes. The size of the accessory genome varied between strains from each of the four species. (**Ai**) For *C. propinquum,* KPL2654 and KPL4040 had the largest accessory sizes, whereas KPL2785 and KPL3672 had the smallest. (**Aii**) For *C. pseudodiphtheriticum,* KPL2640 and MSK188 had the largest accessory sizes, whereas MSK080 and KPL3070 had the smallest. (**Aiii**) For *C. accolens,* ATCC_49726 and KPL2617 had the largest accessory sizes, whereas MSK096 and KPL3970 had the smallest. (**Aiv**) For *C. tuberculostearicum* MSK158 and MSK195 had the largest accessory sizes, whereas MSK207 and MSK074 had the smallest.
