# Supplementary material for: Metabolic capabilities are highly conserved among human nasal-associated Corynebacterium species in pangenomic analyses
Source: mSystems. 2024 Nov 7;9(12):e01132-24. doi: 10.1128/msystems.01132-24 (PMC11651106; doi:10.1128/msystems.01132-24)
Supplement: Figure S5 — Additional data related to Fig. 7 and 8. [file msystems.01132-24-s0006.pdf]

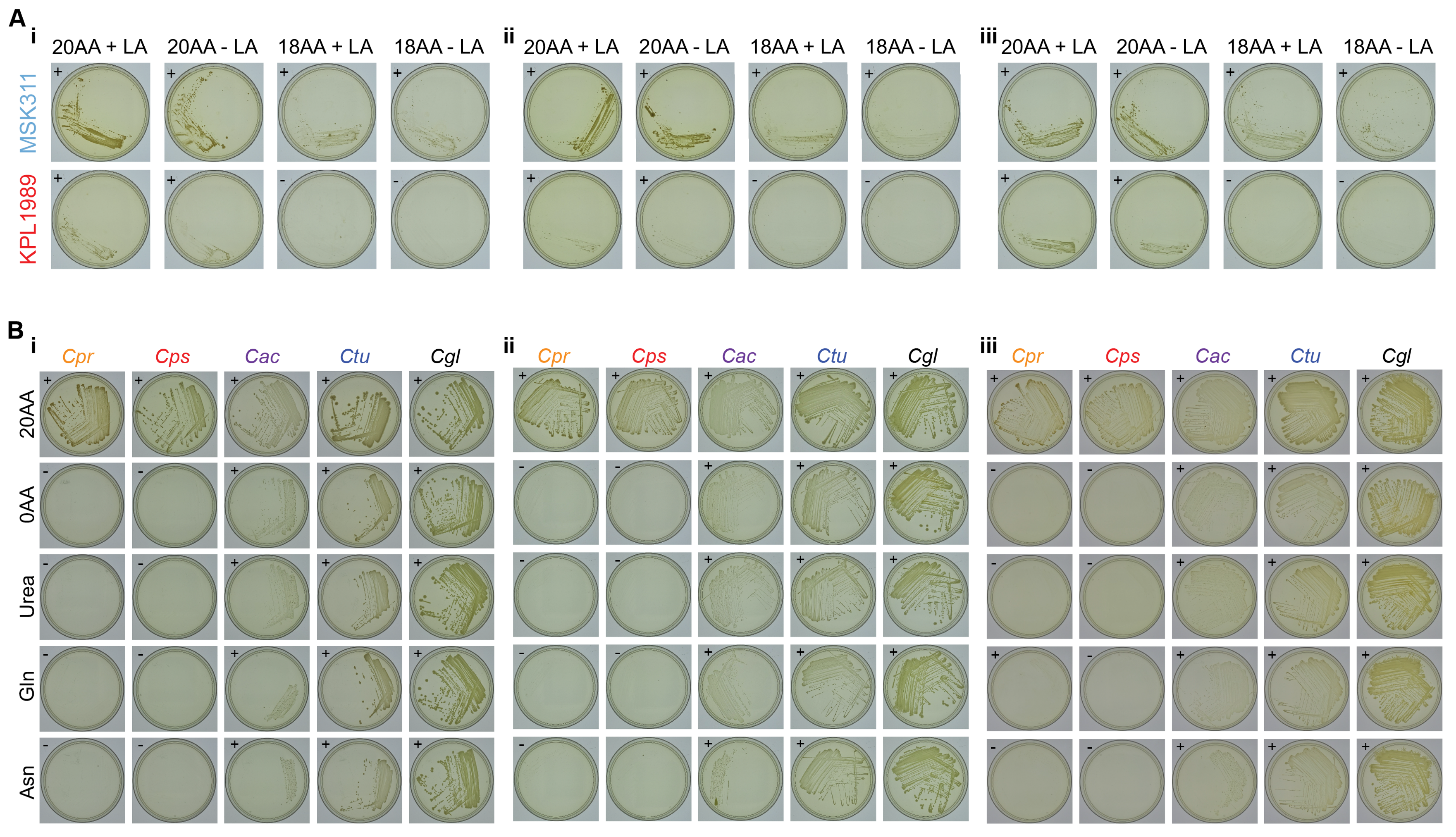

A
i
20AA + LA
20AA - LA
18AA + LA
18AA - LA
ii
20AA + LA
20AA - LA
18AA + LA
18AA - LA
iii
20AA + LA
20AA - LA
18AA + LA
18AA - LA
MSK311
KPL1989
B
i
Cpr
Cps
Cac
Ctu
Cgl
ii
Cpr
Cps
Cac
Ctu
Cgl
iii
Cpr
Cps
Cac
Ctu
Cgl
20AA
0AA
Urea
Gln
Asn

Figure S5. Results of additional growth experiments from Figures 7 (A) and 8 (B). (**A**) The Botswana *C. pseudodiphtheriticum* strain MSK311, which encodes the *fpr2cysIXHDNYZ* operon, grew on chemically defined agarose medium with sulfate as the only source of sulfur (KS-CDM 18AA - LA), whereas the USA strain KPL1989, which lacks this operon, did not. (**B**) *C. accolens* KPL1818 (*Cac*) and *C. tuberculostearicum* MSK074 (*Ctu*), as well as *C. glutamicum* DSM 20300^T (*Cgl*), grew on MOPS-buffered CDM agarose medium in the absence of all 20 amino acids, whereas *C. propinquum* KPL3953 (*Cpr*) and *C. pseudodiphtheriticum* MSK311 (*Cps*) did not. All images were captured after 8-9 days of growth at 34°C with 5% CO_2 with a humidification pan, with all plates from a single experiment imaged on the same day. (+) = growth, (-) = no growth.
